# Dietary Bioactive Lipid Compounds Rich in Menthol Alter Interactions Among Members of Ruminal Microbiota in Sheep

**DOI:** 10.3389/fmicb.2019.02038

**Published:** 2019-09-04

**Authors:** Amlan K. Patra, Tansol Park, Hannah-Sophie Braun, Sebastian Geiger, Robert Pieper, Zhongtang Yu, Jörg R. Aschenbach

**Affiliations:** ^1^Institute of Veterinary Physiology, Freie Universität Berlin, Berlin, Germany; ^2^Department of Animal Nutrition, West Bengal University of Animal and Fishery Sciences, Kolkata, India; ^3^Department of Animal Sciences, The Ohio State University, Columbus, OH, United States; ^4^PerformaNat GmbH, Berlin, Germany; ^5^Institute of Animal Nutrition, Freie Universität Berlin, Berlin, Germany

**Keywords:** correlation network analysis, microbiota composition, menthol, ruminal fermentation, sheep

## Abstract

This study aimed to investigate the effects of two practically relevant doses of menthol-rich plant bioactive lipid compounds (PBLC) on fermentation, microbial community composition, and their interactions in sheep rumen. Twenty-four growing Suffolk sheep were divided into three treatments and were fed hay *ad libitum* plus 600 g/d of concentrate containing no PBLC (Control) or PBLC at low dose (80 mg/d; PBLC-L) or high dose (160 mg/d; PBLC-H). After 4 weeks on the diets, samples of ruminal digesta were collected and analyzed for short-chain fatty acid (SCFA), ammonia, and microbiota; microbiota being analyzed in the solid and the liquid digesta fractions separately. Ruminal SCFA and ammonia concentrations were not affected by the PBLC treatments. The microbiota in the solid fraction was more diverse than that in the liquid fraction, and the relative abundance of most taxa differed between these two fractions. In the solid fraction, phylogenetic diversity increased linearly with increased PBLC doses, whereas evenness (lowest in PBLC-L) and Simpson diversity index (greatest in PBLC-H) changed quadratically. In the liquid fraction, however, the PBLC supplementation did not affect any of the microbial diversity measurements. Among phyla, *Chloroflexi* (highest in PBLC-L) and unclassified_bacteria (lowest in PBLC-L) were altered quadratically by PBLC. *Lachnospiraceae*, *Bacteroidaceae* (increased linearly), *BS11* (increased in PBLC-L), *Christensenellaceae* (decreased in PBLC treatments), and *Porphyromonadaceae* (increased in PBLC treatments) were affected at the family level. Among genera, *Butyrivibrio* increased linearly in the solid fraction, *YRC22* increased linearly in the liquid fraction, whereas *Paludibacter* increased and *BF311* increased linearly with increasing doses of PBLC in both fractions. The PBLC treatments also lowered methanogens within the classes *Thermoplasmata* and *Euryarchaeota*. Correlation network analysis revealed positive and negative correlations among many microbial taxa. Differential network analysis showed that PBLC supplementation changed the correlation between some microbial taxa and SCFA. The majority of the predicted functional features were different between the solid and the liquid digesta fractions, whereas the PBLC treatments altered few of the predicted functional gene categories. Overall, dietary PBLC treatments had little influence on the ruminal fermentation and microbiota but affected the associations among some microbial taxa and SCFA.

## Introduction

Phytobiotics or plant bioactive molecules have been considered a new class of feed additives for livestock and poultry because of their several beneficial responses in animal production (Van Bibber-Krueger et al., 2016; Kumar et al., 2018; Szczechowiak et al., 2018). Depending upon the type and amount of bioactive compounds used, beneficial biological effects may include modulation of ruminal fermentation (Calsamiglia et al., 2007; Mirzaei-Alamouti et al., 2016; Kazemi-Bonchenari et al., 2018), inhibition of methane production and protein degradation (Cobellis et al., 2016; Patra et al., 2017; Soltan et al., 2018), decreased growth of pathogenic microorganisms in the intestines, boosting of immunity, augmentation of antioxidant activities in the animal tissues (Chowdhury et al., 2018; Kumar et al., 2018), regulation of gastrointestinal nutrient transport and barrier function (Patra et al., 2018), improvement of growth performance (Kazemi-Bonchenari et al., 2018) and body condition (Hausmann et al., 2017, 2018), and increased quantity and quality of milk and meat (Hausmann et al., 2018; Smeti et al., 2018). Based on their molecular structures, these secondary plant compounds can be separated into several classes, namely, saponins, tannins, flavonoids, alkaloids, organosulfur compounds, terpenoids, and phenylpropanoids (Wink, 2003; Patra, 2012). The so-called “essential oils” as a very important group of plant bioactive lipid compounds (PBLC) have been investigated widely in the diets of livestock and poultry due to their antimicrobial properties and specificity to certain microbes (Patra, 2011; Cobellis et al., 2016; Chowdhury et al., 2018). In ruminants, PBLC could inhibit some undesirable microbes, such as methanogenic archaea and protein-degrading bacteria, thus improving ruminal fermentation (McIntosh et al., 2003; Patra and Saxena, 2010; Cobellis et al., 2016).

In several previous trials, high doses of menthol-rich peppermint oil decreased methane production and altered the concentrations and proportions of short chain fatty acid (SCFA) in the ruminal fluid *in vitro* (up to 2 mL/L, Agarwal et al., 2009; up to 1 g/L, Patra and Yu, 2012; up to 0.6 g/L, Roy et al., 2015). In these *in vitro* studies, few select microbial populations (e.g., *Ruminococcus* spp., *Fibrobacter succinogenes*, methanogenic archaea, and protozoal numbers) were specifically affected by the menthol supplementation (Agarwal et al., 2009; Patra and Yu, 2012). In contrast, peppermint herbs fed at dosages of 50 g/kg DM to lactating Holstein cows (Hosoda et al., 2005) or at 200 g/d in steers (Ando et al., 2003) had no effect on the total concentration and molar proportions of SCFA. Assuming a menthol content of the applied peppermint supplements of ∼1% (Beigi et al., 2018), the menthol doses used in the latter *in vivo* studies were far lower than those used *in vitro*, however, they were yet higher than the doses that can be used economically and safely in routine practical feeding. Regarding safety, current EU regulations acknowledge that menthol supplementation is safe up to a concentration of 25 mg/kg complete feeding stuff and require detailed labeling where this concentration is exceeded (The European Commission, 2018).

The ruminal microbiota is highly dynamic and has diverse microbes associated with ruminal epithelium, digesta solids, or ruminal liquid (Mao et al., 2015; Klevenhusen et al., 2017). The three fractions have distinctive microbiota diversity, composition, and functions and perform different ecological and nutritional roles (Mao et al., 2015; Klevenhusen et al., 2017). It has been shown repeatedly that dietary PBLC including menthol can modulate ruminal fermentation by exerting antimicrobial actions (Calsamiglia et al., 2007; Patra and Saxena, 2009; Khiaosa-ard and Zebeli, 2013). To the best of our knowledge, however, no study has investigated the effects of any PBLC on microbiota in the different digesta fractions separately. We thus hypothesized that menthol might affect the microbiota in the solid and the liquid phase differently. Moreover, considering the specific antimicrobial actions of high doses of peppermint oil that lead to fermentation changes *in vitro* vs. an apparent absence of fermentation changes when menthol is supplemented at lower doses *in vivo*, it was further hypothesized that menthol effects on specific microbial populations may not result in detectable fermentation changes *in vivo* due to compensatory alterations in metabolic interactions among microbial species. To test the above hypotheses, this study was performed to investigate the effects of menthol-rich PBLC at two doses with practical relevance and yet justifiable safety on ruminal fermentation and its relation to the composition, structures, functions and interactions of microbiota in the solid and liquid fractions of ruminal digesta.

## Materials and Methods

### Experimental Design, Animals, and Feeding

Twenty-four growing Suffolk sheep (15 females and 9 males) with initial body weight (BW) of 32.9 ± 3.44 kg and age of 121 ± 3.75 days were equally distributed into three experimental groups that either received no PBLC (Control; *n* = 8), 80 mg PBLC/d (PBLC-L; *n* = 8) or 160 mg PBLC/d (PBLC-H; *n* = 8). The experiment was performed in two feeding experimental runs with 12 sheep in the first run and 12 different sheep in the second run. Sheep were distributed to the three dietary treatment groups (Control, PBLC-L and PBLC-H) with the aim to achieve equal sex (5 females and 3 males, per group) and BW distribution by using a randomized block design. Each block consisted of one sheep per group with similar initial BW. This resulted in a total of four blocks of three sheep per run, which were accommodated to the four available pens. Each pen contained three separate automatic transponder-operated feeding stations with locking gates designed for small ruminants. Each sheep had access to only one specific feeder recognized by individual animal identification tag with an electronic transponder fitted to its neck collar.

Based on an (National Research Council [NRC], 2007) requirement for 300 g/d growth rate, all sheep were fed a pelleted concentrate (600 g/d) and *ad libitum* meadow hay (without chopping) for 4 weeks. Ingredients and chemical compositions of concentrates were the same except that the concentrates of the PBLC-L and PBLC-H groups were pelleted together with a PBLC premix (OAX17, PerformaNat GmbH, Germany; 13.33 g PBLC/kg of ground corn grains) to achieve the final target PBLC concentrations (133 or 267 mg/kg concentrate) (Table 1). Concentrates were pelleted below 50°C to avoid evaporative loss of PBLC during pelleting, and the concentrate pellets were stored in air-tight bags. The daily dose of PBLC was supplied with the concentrates at 07:00, 11:00, and 15:00 h in three equal portions (200 g each time). All sheep completely consumed the concentrate feed allowance. The commercial source of PBLC contained mainly menthol (900 g/kg) with other minor bioactive compounds. Drinking water was available at all times from push-button water troughs. Feed samples (hay and concentrate mixtures) were collected weekly and pooled for each treatment for chemical analyses.

**TABLE 1:** Ingredient and chemical composition of pelleted concentrates and hay fed to sheep.

	**Concentrate^a^**			**Hay**
		
	**Control**	**PBLC-L**	**PBLC-H**	
**Ingredient composition (g/kg)**				
Corn	305	295	285	
Barley	305	305	305	
Soybean meal	348	348	348	
Molasses	30	30	30	
Mineral and vitamin premix^b^	5	5	5	
Salt	2	2	2	
Limestone	5	5	5	
Additive premix^c^	0	10	20	
**Chemical composition**				
Dry matter (DM; g/kg as-fed)	914	915	912	923
Organic matter (g/kg DM)	949	950	948	958
Crude protein (g/kg DM)	259	257	259	108
Ether extract (g/kg DM)	30.8	25.1	25.8	9.60
Neutral detergent fiber (g/kg DM)	125	140	152	641
Acid detergent fiber (g/kg DM)	65.4	74.6	70.7	374
Crude fiber (g/kg DM)	48.9	41.7	43.0	332
Calcium (g/kg DM)	5.31	5.36	5.83	3.11
Phosphorus (g/kg DM)	5.09	4.81	4.74	2.08
Metabolizable energy (MJ/kg DM)^d^	12.6	12.6	12.6	7.53

**^*a*^Control: without PBLC; PBLC-L: low dose (133 mg/kg feed) of PBLC; and PBLC-H: high dose (267 mg/kg feed) of PBLC. ^*b*^Mineral and vitamin premix (Spezialfutter Neuruppin Ltd., Neuruppin, Germany), containing per kg dry matter: 160 g calcium, 40 g phosphorus, 100 g sodium, 30 g magnesium, 500,000 IU vitamin A, 50,000 IU vitamin D3, 500 mg vitamin E (as alpha-tocopherol acetate), 4500 mg zinc (as zinc oxide), 500 mg manganese [as Mn-(II)-oxide], 20 mg cobalt [as Co-(II)-carbonate], 20 mg iodine (as calcium iodate), and 35 mg selenium (as sodium selenite). ^*c*^Additive premix contained 13.33 g PBLC/kg of ground corn grains. ^*d*^Calculated from individual ingredients (National Research Council [NRC], 2007).**

### Feed Analysis

The chemical composition of feed samples was analyzed following standard methods (Naumann et al., 2004). Briefly, content of dry matter was determined in a drying cabinet (VDLUFA MB III 3.1), crude ash in a muffle furnace at 550°C (VDLUFA III 8.1), ether extract by the Soxhlet method with hydrolysis (VDLUFA MB III 5.1.1), and crude protein by the incineration method (VDLUFA MB III 4.1.2). Acid detergent fiber (ADF) expressed exclusive of residual ash (ADF_om_) was determined using FibertecTM 8000 (FOSS, Hilleroed, Denmark; method, VDLUFA MB III 6.5.2). Neutral detergent fiber (NDF) content (comparable to the assay with heat-stable amylase and expressed exclusive of residual ash; aNDF_om_) was estimated using near-infrared spectroscopy according to the method VDLUFA MB III 31.2.

### Sampling of Ruminal Solid and Liquid Fractions and Microbiota Analysis

After the 4 weeks trial period, sheep were maintained on the assigned feeding regime until slaughter using penetrative captive bolt with subsequent exsanguination. Animals from the three groups were killed in the order of their block designation (i.e., block after block) over 6 consecutive days. Each day, one animal was killed 2 h after the morning feeding and a second animal was killed 2 h after the 11:00 h feeding. As group order of killing was kept constant for all blocks, exactly half of the animals in each group had been slaughtered 2 h after the morning feeding and the other half had been slaughtered 2 h after the 11:00 h feeding at the end of the trial. The pH in the ventral sac of the rumen was recorded immediately after killing using pH meter 3110 (Xylem Analytics Germany GmbH, Weilheim, Germany). One ruminal content sample of each sheep was filtered through two layers of sterile cheesecloth to separate solid and liquid fractions for microbiota analysis. These samples were immediately placed on ice and stored within 30 min at −20°C until DNA extraction. To determine the concentrations of short-chain fatty acids (SCFA) and ammonia, an aliquot of 8 mL of ruminal fluid from each sheep was immediately transferred into a 15 mL polypropylene centrifuge tube (TPP^®^, Trasadingen, Switzerland) containing 2 mL of metaphosphoric acid solution (250 g/L distilled water) to precipitate protein and stop fermentation. The tubes were kept at room temperature for 30 min and then placed on ice. The ruminal fluid was centrifuged at 5000 × *g* at 4°C for 15 min and the supernatant was stored at −20°C until analysis. Concentrations of SCFA (acetic, propionic, *n*-butyric, *iso*-butyric, *n*-valeric, and *iso*-valeric acids) and ammonia were analyzed as described by Pieper et al. (2014). Methane production was estimated from the stoichiometric relations between SCFA concentrations and methanogenesis (Moss et al., 2000).

### Extraction of DNA

Metagenomic DNA from the solid and liquid fractions of the rumen samples was extracted using the repeated bead beating (with a Homogenizer MM301, Retsch, Haan, Germany at a frequency up to 30 Hz) plus column purification method as described by Yu and Morrison (2004). The quality of extracted DNA was assessed based on the absorbance ratios of 260/280and 260/230 nm and quantified using a NanoDrop ND-2000 Spectrophotometer (Thermo Scientific, Wilmington, DE, United States). The DNA samples were dried under vacuum and sent to the DNA sequencing facility at The Ohio State University (United States) and stored at −20°C until analysis.

### 16S rRNA Gene Sequencing and Downstream Analysis

Amplicon libraries of 16S rRNA genes were prepared and sequenced at the Molecular and Cellular Imaging Center of The Ohio State University^1^. Briefly, dual index 16S rRNA gene amplicon libraries from both bacteria and archaea were prepared following the Illumina protocol (Fadrosh et al., 2014; Illumina 16S Metagenomic Sequencing Library Preparation guide – Part # 15044223 Rev. B) using 515F (5′-GTGCCAGCMGCCGCGGTAA-3′) and 806R (5′-GGACTACHVGGGTWTCTAAT-3′) specific primers (Caporaso et al., 2011) with a unique barcode for each DNA sample. The amplicon libraries were pooled equimolarly and sequenced on the MiSeq instrument in a 300 cycles paired-end run. The built-in plugins within QIIME2 (version 2018.6) (Bolyen et al., 2018) were used to analyze the 16S rRNA amplicon sequences. Briefly, DADA2 was first used to denoise the demultiplexed forward and the reverse reads with quality (Q > 25) filtering (Callahan et al., 2016). The denoised-feature table and amplicon sequence variants (ASVs) were used for taxonomic diversity analysis. The ASVs were taxonomically classified with the Greengenes 99% OTUs 16S rRNA gene reference sequences (version 13_8) using the naïve Bayesian taxonomic classifier (Wang et al., 2007). The relative abundance of a taxon was expressed as percentage of total sequences in respective samples. Assigned taxa were visualized through bar plots using the mean relative abundance of each group. Mitochondria sequences were removed in the final analysis. Raw 16S rRNA gene amplicon sequence data are available in NCBI Sequence Read Archive (SRA) under BioProject PRJNA529255.

### Prediction of Metabolic Pathways and Functions of Microbiota

The functional features of each sample were predicted using PICRUSt (Phylogenetic Investigation of Communities by Reconstruction of Unobserved States) (Langille et al., 2013). Species-level OTUs were picked using the q2-vsearch closed-reference OTU picking method against the Greengenes reference database of 16S rRNA gene sequences clustered at 97% sequence similarity (13_5 release). Copy number-based normalized classified OTUs table was used to link in between Greengenes IDs and KEGG orthologs by finding genome contents for each OTU from pre-calculated file followed by multiplying the OTU abundance by predicted functional abundance in the genome using PICRUSt.

### Statistical Analysis

The Mixed model procedure of SAS (2001) was used to analyze the relative abundance of phylum, family and genus, and KEGG gene abundance at subsystem levels 1, 2, and 3. Residuals were checked for normality using either Shapiro–Wilk or Kolmogorov–Smirnov test. When residuals did not show normal distribution, data were transformed (log, arcsine, sine, or square-root) to have normal distribution. If residuals did not follow normal distribution after various transformations, the non-parametric Wilcoxon test was used to compare solid vs. liquid fractions and Control vs. both PBLC treatments. Unequal variances among treatments, if any, were adjusted using the “Repeated” statement in the mixed model. The relative abundances of all the identified phyla and KEGG level 1 gene categories were analyzed, while only the relative abundance of the major families, genera, KEGG subsystem level 2 and level 3 categories (each with a relative abundance >0.5%) were comparatively analyzed because of the large numbers of taxa and functional categories identified. The model included treatment, block (i.e., initial BW of sheep), fractions of digesta, sex and fraction × treatment interaction. Linear and quadratic effects of PBLC doses (0, 80, and 160 mg/d) were assessed using polynomial contrasts. Contrasts between Control (0 mg/d of PBLC) vs. average of both PBLC groups (80 and 160 mg/d of PBLC) were also used to determine the overall effects of PBLC compared with the Control. When interaction was or tended to be significant, the SLICE option in the model was used to determine the effect in either the solid or liquid fraction. Variability of data was expressed as pooled SEM, and statistical significance was set at *P* ≤ 0.05, while a trend was considered at 0.05 < *P* ≤ 0.10.

Principal component analysis (PCA) was performed to investigate differences in the predicted functions at KEGG level 3 among the treatments and between the two digesta fractions using PROC PRINCOMP of SAS (2001) with the covariance matrix. The first three principal components were plotted to show the overall comparison of the treatments. The PCA loadings of the first three components were further analyzed using SAS (2001) by multivariate analysis of variance (MANOVA) to test for differences in the overall predicted functional features between digesta fraction, treatment and treatment × fraction interaction. Aided by an online tool^2^, linear discriminant analysis (LDA) effect size (LEfSe) and Kruskal-Wallis tests were used to find differentially abundant (*P* < 0.05 and LDA scores higher than 2; Segata et al., 2011) taxa and functional features.

Pearson correlations between SCFA and major OTUs each with a relative abundance >0.1% and among the OTUs each with a relative abundance >0.5% were calculated using SAS (2001). These cutoffs were used based on the assumption that the small OTUs with a relative abundance below these cutoffs would have minimal influence on the functionality of the entire microbiota. The force-directed correlation network layouts based on the numerical values of correlations coefficients were then created including nodes consisting of microbiota and SCFA pattern which had a *P* ≤ 0.01 using Cytoscape (Shannon et al., 2003). We chose the strong correlation (*P* ≤ 0.01) cutoffs to focus on substantial contributions in the interactions. These networks were created separately for the Control group and the PBLC groups, or the liquid fraction and the solid fraction. Correlation differences were also analyzed to evaluate the differential features between Control and PBLC treatments, and liquid and solid fractions, and these networks were visualized in Cytoscape (Shannon et al., 2003).

## Results

### Ruminal Fermentation

Ruminal fermentation characteristics including pH, total SCFA concentrations and molar proportion of acetate, propionate, butyrate and other minor acids, and acetate to propionate ratio were not affected by the dietary PBLC supplementation (Table 2). Ammonia concentrations in ruminal fluid were not changed by PBLC. The estimated methane production in sheep was also not influenced by PBLC feeding (Table 2).

**TABLE 2:** Effect of two doses of dietary menthol-rich plant bioactive lipid compounds (PBLC) on ruminal pH and short-chain fatty acids (SCFA) in sheep.

**Fermentation characteristics**	**Treatment^a^**			**SEM**	***P*-value**		
			
	**Control**	**PBLC-L**	**PBLC-H**		**Linear**	**Quadratic**	**Control vs. PBLC**
pH	6.16	6.19	6.18	0.042	0.69	0.73	0.60
Total SCFA (mM)	109	100	103	7.3	0.56	0.51	0.40
Acetate (A; mole/100 moles)	68.7	68.6	67.7	0.54	0.23	0.59	0.42
Propionate (P; mole/100 moles)	17.3	16.8	17.8	0.62	0.60	0.29	0.94
Isobutyrate (mole/100 moles)	0.96	1.02	1.02	0.059	0.48	0.67	0.41
Butyrate (mole/100 moles)	11.0	11.5	11.1	0.59	0.85	0.52	0.63
Isovalerate (mole/100 moles)	1.12	1.32	1.22	0.117	0.57	0.33	0.33
Valerate (mole/100 moles)	1.03	1.11	1.08	0.069	0.85	0.51	0.42
A:P ratio	3.99	4.12	3.85	0.116	0.38	0.17	0.96
Ammonia (mmol/L)	13.6	14.2	13.7	1.02	0.92	0.65	0.75
Methane (L/mole of SCFA)^b^	6.83	6.91	6.72	0.074	0.29	0.15	0.87

**^*a*^Control, PBLC-L, and PBLC-H, treatment groups supplemented with menthol-rich PBLC at 0, 80, and 160 mg/d, respectively. ^*b*^Estimated from stoichiometric relations, methane = 0.45 × acetate – 0.275 × propionate + 0.40 × butyrate (Moss et al., 2000).**

### Rumen Microbiota Diversity

On average, 37,359 quality checked sequences were obtained from each sample, which allows for >99% depth coverage. As shown in Table 3, all the determined alpha diversity measurements, except for Simpson diversity index (*P* = 0.103) and evenness (*P* = 0.69), were greater in the solid fraction than in the liquid fraction. In the solid fraction, the number of observed OTU and Chao 1 richness estimate were not affected (*P* > 0.10) by PBLC, but phylogenetic diversity (*P* = 0.028) increased linearly with PBLC doses; whereas evenness (lowest in PBLC-L), and Simpson diversity index (greatest in PBLC-H) were quadratically changed by PBLC (*P* = 0.006–0.001). In the liquid fraction, however, none of the determined alpha diversity measurements was influenced by the PBLC supplementation (*P* > 0.10) except for Simpson index (increased linearly, *P* = 0.094). Interactions between treatment and digesta fraction were not significant (*P* > 0.10) for any of the alpha diversity measurements.

**TABLE 3:** Effect of dietary menthol-rich plant bioactive lipid compounds (PBLC) on ruminal microbiota diversity measurements in the solid and the liquid fractions of sheep ruminal digesta.

**Diversity measurements**	**Treatment^a^**			**SEM**	***P*-value**		
			
	**Control**	**PBLC-L**	**PBLC-H**		**Linear**	**Quadratic**	**Control vs. PBLC**
**Solid fraction^b^**							
Observed OTUs^c^	74.3	76.1	81.3	3.18	0.14	0.68	0.27
Chao1	75.9	77.3	82.8	3.37	0.16	0.61	0.33
Coverage (%)	99.9	99.9	99.8	0.02	0.016	0.16	0.13
Phylogenetic diversity	35.3	36.0	40.1	1.39	0.028	0.32	0.12
Evenness	0.868	0.843	0.868	0.0052	0.90	0.001	0.076
Shannon	7.85	7.70	8.06	0.127	0.25	0.12	0.84
Simpson^d^	0.988	0.984	0.990	0.0013	0.22	0.006	0.65
**Liquid fraction^b^**							
Observed OTUs^c^	63.4	62.9	64.6	3.77	0.83	0.82	0.95
Chao1	63.9	63.4	65.2	3.97	0.81	0.82	0.93
Coverage (%)	99.9	99.9	99.9	0.02	0.74	0.57	0.57
Phylogenetic diversity	28.8	28.3	30.4	2.18	0.60	0.64	0.83
Evenness	0.863	0.863	0.873	0.0068	0.28	0.57	0.51
Shannon	7.21	7.23	7.47	0.144	0.22	0.56	0.44
Simpson^d^	0.984	0.984	0.988	0.0018	0.094	0.19	0.43

**^*a*^Control, PBLC-L, and PBLC-H, treatment groups supplemented with menthol-rich PBLC at 0, 80, and 160 mg/d, respectively.****^*b*^****Correlation coefficient for OTUs between the solid and the liquid fractions was 0.36 (P = 0.083). Treatment × fraction was not significant (P > 0.10) for all the measurement except coverage (P = 0.050). There were significant differences between the solid and the liquid fractions for all the diversity measurements except Simpson (P = 0.103) and evenness (P = 0.69). ^*c*^OTUs were clustered based on 99% sequence similarity. ^*d*^Data were sine-transformed to achieve normality. Observed OTUs and Chao1 observes and estimates the number of OTUs, respectively. Good’s coverage estimates the percentage of entire species in a sample. Phylogenetic diversity measures phylogenetic difference between detected species by summing the length of branches. Evenness represents the relative evenness of species richness. Shannon explains both abundance and evenness of detected taxa. Simpson measures the number and abundance of detected taxa.**

### Microbiota Composition

In the solid fraction, 104 species-level OTUs were shared among all the treatments, but 5, 7, and 11 OTUs were unique to the Control PBLC-L, and PBLC-H, respectively. In the liquid fraction, the number of shared OTUs was 93, whereas 7, 11, and 6 OTUs were unique to Control, PBLC-L and PBLC-H, respectively (Supplementary Figure S1).

**FIGURE 1:**
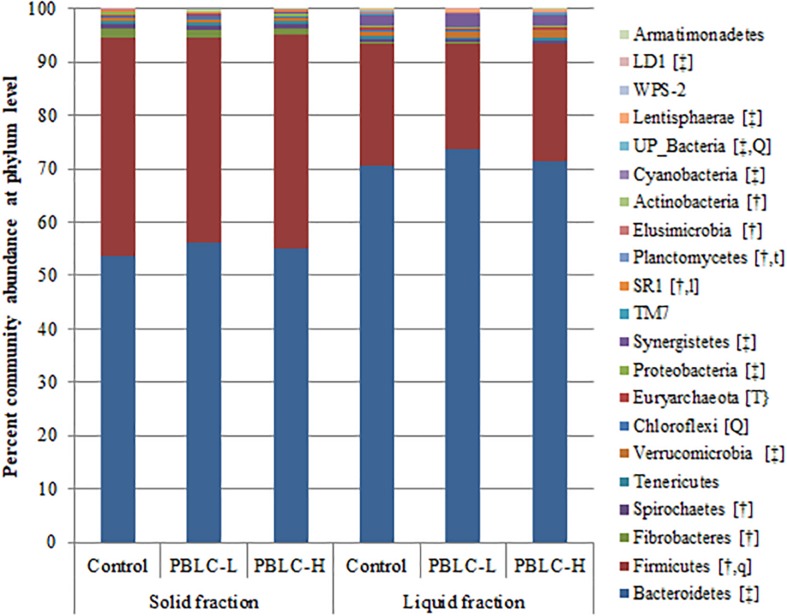
Community barplot analysis depicting the average relative abundance of phyla detected in solid and liquid fractions of the ruminal digesta of different treatment groups. Control, PBLC-L, and PBLC-H, treatment groups supplemented with menthol-rich plant bioactive lipid compounds at 0, 80, and 160 mg/d, respectively. In the square brackets, symbols † and ‡ indicate greater (*P* ≤ 0.05) abundances in the solid and the liquid fractions, respectively, while uppercase letters indicate significant (*P* ≤ 0.05) treatment effect (T; Control vs. both PBLC-L and PBLC-H) or dose effect (L for linear, Q for quadratic) of PBLC; whereas, lowercase letters (t for treatment, and l and q for linear and quadratic dose effect, respectively) indicate a trend (0.05 < *P* ≤ 0.10). No interactions between treatment and digesta fraction were present (*P* ≥ 0.10).

Twenty-one different phyla were identified in all the samples, and all differed in relative abundance between the solid and liquid fractions, except for *Tenericutes, Chloroflexi, Euryarchaeota, Proteobacteria, TM7, WPS-2*, and *Armatimonadetes*. *Firmicutes, Fibrobacteres, Spirochetes, SR1, Planctomycetes*, and *Actinobacteria* were more predominant (*P* ≤ 0.05) in the solid fraction than in the liquid fraction, while *Bacteroidetes, Verrucomicrobia, Synergistetes, Cyanobacteria*, *Lentisphaerae*, and *LD1* showed an opposite distribution (*P* ≤ 0.05) (Figure 1 and Supplementary Table S1). Interaction effects between treatment and digesta fraction were not evident (*P* ≥ 0.10) for any of the phyla. Among the phyla, *Firmicutes* (quadratic response; *P* = 0.065; lowest in PBLC-L), *Chloroflexi* (quadratic response; *P* = 0.004; highest in PBLC-L), *Planctomycetes* (decreased in both PBLC treatments vs. Control; *P* = 0.062), UP_bacteria (quadratic response; *P* = 0.007; lowest in PBLC-L), *SR1* (tended to increase linearly; *P* = 0.051), and *Euryarchaeota* (decreased in both PBLC treatments vs. Control; *P* = 0.009) were influenced by the PBLC treatments.

**FIGURE 2:**
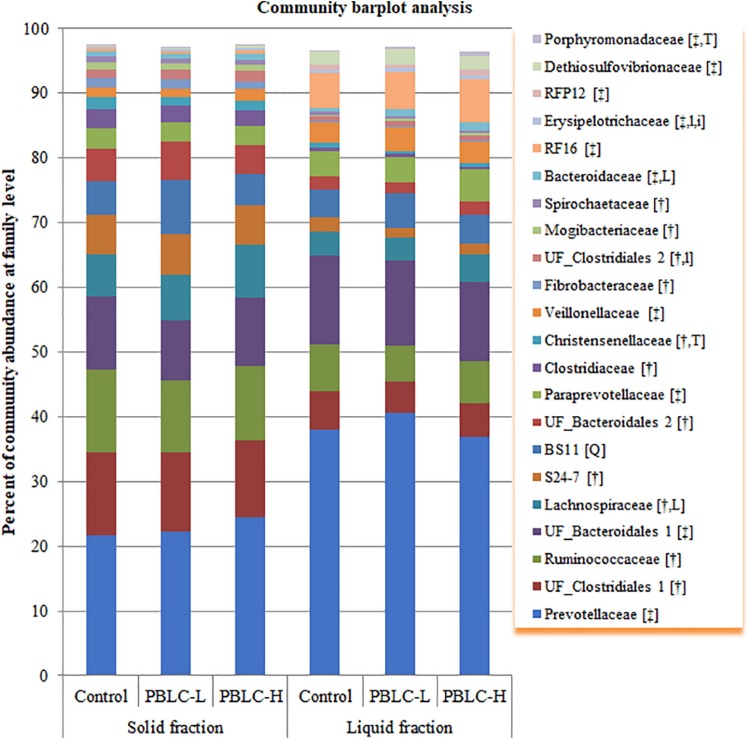
Community barplot analysis depicting the average relative abundance of the major families (each representing ≥0.5% total sequences) detected in solid and liquid fractions of the rumen digesta of different treatment groups. Control, PBLC-L, and PBLC-H, treatment groups supplemented with menthol-rich plant bioactive lipid compounds at 0, 80, and 160 mg/d, respectively. In the square brackets, symbols † and ‡ indicate grater (*P* ≤ 0.05) abundances in the solid and the liquid fractions, respectively, while uppercase letters indicate significant (*P* ≤ 0.05) treatment effect (T; Control vs. both PBLC-L and PBLC-H) or dose effect (L for linear, Q for quadratic) of PBLC; whereas, lowercase letters (t for treatment, and l and q for linear and quadratic dose effect, and i for interaction effect between treatment and digesta fraction) indicate a trend (0.05 < *P* ≤ 0.10).

Ninety-one different families were found from at least one sample, and 21 families including four unclassified families (UF) had a relative abundance of >0.5% in at least one of the fractioned samples. All these major families were significantly different (*P* = 0.03 to < 0.001) in relative abundance between the solid and the liquid fractions, except for *BS11* (*P* = 0.10) (Figure 2 and Supplementary Table S2). Overall, the solid fraction had a greater relative abundance of UF_*Clostridiales* 1, *Ruminococcaceae, Lachnospiraceae, S24-7*, UF_*Bacteroidales* 2, *Clostridiaceae, Christensenellaceae, Fibrobacteraceae*, UF_*Clostridiales* 2, *Mogibacteriaceae*, and *Spirochaetaceae*, but a lower relative abundance of *Prevotellaceae*, UF*_Bacteroidales* 1, *Paraprevotellaceae, Veillonellaceae, Bacteroidaceae, RF16, Erysipelotrichaceae, RFP12, Dethiosulfovibrionaceae*, and *Porphyromonadaceae* than the liquid fraction (*P* ≤ 0.05). *Lachnospiraceae* (*P* = 0.019), *Bacteroidaceae* (*P* = 0.026), and UF_*Clostridiales* 2 (*P* = 0.060) tended to increase or increased linearly with increasing doses of PBLC. A trend in fraction × treatment interaction (*P* = 0.075) was noted for *Erysipelotrichaceae*, which was not affected by the PBLC treatments in the solid fraction but increased linearly in the liquid fraction. *BS11* increased quadratically (*P* = 0.012) with the highest relative abundance in the PBLC-L, while *Christensenellaceae* decreased (*P* = 0.010) and *Porphyromonadaceae* increased (Wilcoxon test, *P* = 0.008) in both PBLC groups.

**FIGURE 3:**
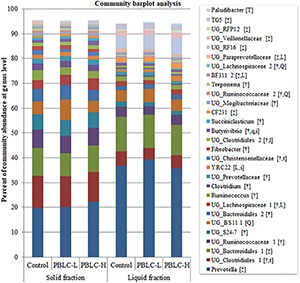
Community barplot analysis depicting the average relative abundance of the major genera (each representing ≥0.5% total sequences) detected in the solid and the liquid fractions of the rumen digesta of different treatment groups. Control, PBLC-L, and PBLC-H, treatment groups supplemented with menthol-rich plant bioactive lipid compounds at 0, 80, and 160 mg/d, respectively. In the square brackets, symbols † and ‡ indicate greater (*P* ≤ 0.05) abundances in the solid and the liquid fractions, respectively, while uppercase letters indicate significant (*P* ≤ 0.05) treatment effect (T; Control vs. both PBLC-L and PBLC-H) or dose effect (L for linear, Q for quadratic) of PBLC; whereas, lowercase letters (t for treatment, and l and q for linear and quadratic dose effect, and i for interaction effect between treatment and digesta fraction) indicate a trend (0.05 < *P* ≤ 0.10).

The present study identified 144 genera in at least one sample, and many of them were unclassified genera (UG). Twenty-eight genera had relative abundances >0.5%, and all these genera significantly differed in relative abundance between the solid and the liquid fractions (*P* = 0.03 to < 0.001), except for UG_*BS11* (*P* = 0.10), *YRC22* (*P* = 0.76), and *Paludibacter* (*P* = 0.14) (Figure 3 and Supplementary Table S3). *Ruminococcus, Clostridium, Fibrobacter, Butyrivibrio, Succiniclasticum, Treponema*, several unclassified genera within the families *Ruminococcaceae, S24-7, Lachnospiraceae, Prevotellaceae, Christensenellaceae*, and *Mogibacteriaceae*, or orders *Clostridiales* and *Bacteroidales* were more predominant in the solid fraction than in the liquid fraction (*P* ≤ 0.05), whereas *Prevotella, CF231, BF311, TG5*, UG_*Bacteroidales* 1, UG_*Paraprevotellaceae*, UG_*RF16*, UG_*Veilonellaceae*, and UG_R*FP12* were more predominant in the liquid fraction than in the solid fraction (*P* ≤ 0.05).

Trends for interaction effects between treatment × digesta fraction were noted for the relative abundance of *YRC22* (*P* = 0.077) and *Butyrivibrio* (*P* = 0.088). With the increased doses of PBLC, *Butyrivibrio* increased their relative abundance linearly (*P* = 0.015) in the solid fraction, while *YRC22* did the same (*P* = 0.007) but in the liquid fraction. The relative abundance of UG_*Clostridiales* 1 tended to decrease (*P* = 0.098) and that of UG_*Christensenellaceae* (Wilcoxon *P* = 0.080) and UG_*Paraprevotellaceae* (*P* = 0.036) tended to decrease or decreased linearly in response to the PBLC supplementation irrespective of fractions. On the contrary, *Paludibacter* increased (Wilcoxon test, *P* = 0.043) and the relative abundances of three other taxa (UG_*Lachnospiraceae* 1, *P* = 0.018; *BF311*, *P* = 0.025; and UG_*Clostridiales* 2, *P* = 0.057) tended to increase linearly or increased linearly with increased PBLC doses in both fractions. The PBLC treatments quadratically affected the relative abundance of UG_*BS11* (*P* = 0.012; highest in PBLC-L), UG_*Ruminococcaceae* 2 (*P* = 0.047; lowest in PBLC-L), and UG_*Lachnospiraceae* 2 (*P* = 0.050; lowest in PBLC-L).

**FIGURE 4:**
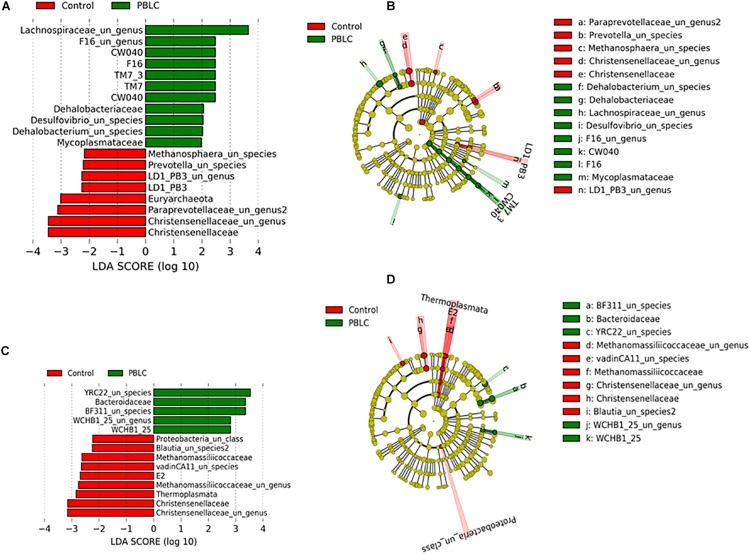
Linear discriminant analysis effect size (LEfSe) and their cladograms of biomarker genera and species in the solid **(A,B)** or liquid **(C,D)** fractions of the rumen digesta. Nodes represent position of microbiota in taxa with circular and axial lines indicate the level of taxa and number of taxa at the lower level. Control and PBLC, sheep supplemented with 0 and 80 or 160 mg/d menthol-rich plant bioactive lipid compounds.

LEfSe analysis showed that some bacteria and archaea were enriched in the solid fraction of the PBLC treatments, including *Dehalobacteriaceae, Mycoplasmataceae*, UG_*Lachnospiraceae*, unclassified species (US)_*Dehalobacterium*, and US_*Desulfovibrio*, whereas *Christensenellaceae*, UG_*Paraprevotellaceae*, *Euryarchaeota*, US_*Methanosphaera*, US_*Prevotella*, *LD1-PB3*, and UG_*LD1-PB3* were significantly more predominant in the solid fraction of the Control than of the PBLC treatments (*P* ≤ 0.05; Figure 4). In the liquid fraction, *WCHB1-25*, US_*WCHB1-25*, *Bacteroidaceae*, US_*BF311*, and US_*YRC22* had a greater relative abundance in the PBLC groups than in the Control, whereas *Christensenellaceae*, UG*_Christensenellaceae, Thermoplasmata*, *Methanomassiliicoccaceae*, and vadinCA11, US_*Blautia* 2 and UC_*Proteobacteria* were significantly greater in the Control than in the PBLC treatments (*P* ≤ 0.05; Figure 4).

### Correlations Between Microbial Taxa and SCFA

**FIGURE 5:**
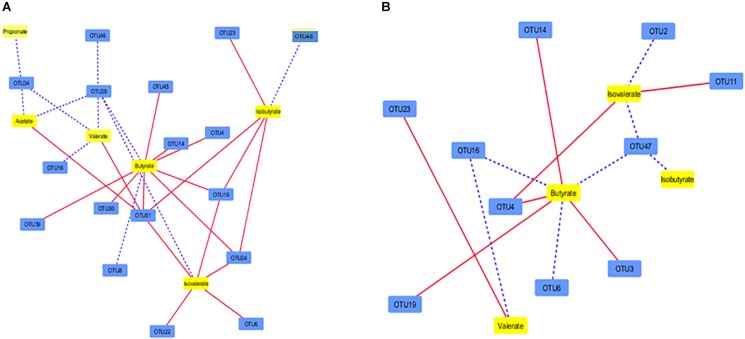
Correlation network showing the correlation between SCFAs in the ruminal fluid and the major OTUs (each representing ≥0.1% total sequences) in the solid **(A)** and liquid fractions of digesta **(B)**. Dotted lines indicate positive correlations whereas solid lines represent negative correlations. The shorter the distances between two nodes, the stronger the correlation between them. See Supplementary Table S5 for the taxonomic assignment of the OTUs.

Irrespective of PBLC supplementation, the concentrations of all six determined SCFAs were correlated (*P* ≤ 0.05) with at least one of the 17 solid-associated OTUs, and there were 29 significant correlations between individual SCFA and OTUs, of which nine were positive (Figure 5A). Only 14 correlations (*P* ≤ 0.05) were found among four SCFAs (all the six SCFA except acetate and propionate) and 10 liquid-associated OTUs, out of which seven were positive (Figure 5B). Between the solid and liquid fractions, an overlap was noted in the correlations of OTU4, OTU14, and OTU19 with butyrate and OTU16 with valerate. Consequently, 25 correlations were unique for the solid-associated OTUs and 10 correlations were unique for the liquid-associated OTUs (Supplementary Figure S2).

**FIGURE 6:**
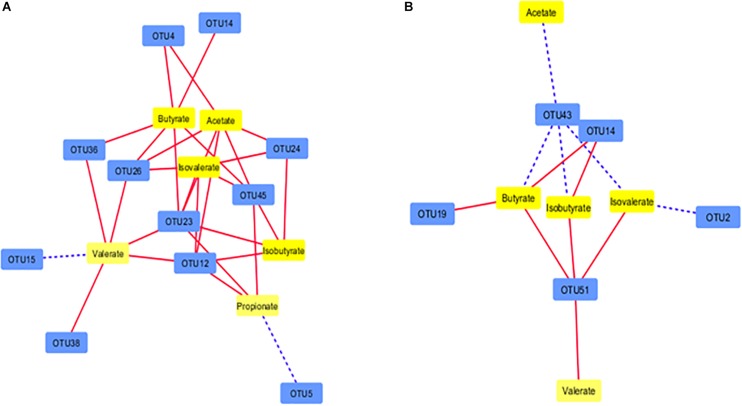
Correlation network showing the correlation between major OTUs (each representing ≥0.1% of total sequences) in the digesta and SCFAs of ruminal fluid in the Control group **(A)** and both PBLC-L and PBLC-H groups **(B)**. Control, PBLC-L, and PBLC-H, treatment groups supplemented with menthol-rich PBLC at 0, 80, and 160 mg/d, respectively. Dotted lines indicate positive correlations, whereas solid lines represent negative correlations. The shorter the distance between two nodes, the stronger the correlation between them. See Supplementary Table S5 for the taxonomic assignment of the OTUs.

In the Control group, 11 OTUs from both digesta fractions were correlated with the concentration of at least one of the six detected SCFAs (9 OTUs negatively and 2 OTUs positively; *P* ≤ 0.05). A total of 31 individual correlations were noted within the correlation network, out of which 29 correlations were negative and two (US*_BS11* with propionate and US_*CF231* with valerate) were positive (Figure 6A). In the PBLC groups, five OTUs from both digesta fractions were associated (*P* ≤ 0.05) to at least one of five SCFAs (all the detected SCFAs except propionate), and of the 12 correlations, seven were negative (Figure 6B). Although PBLC did not influence the concentrations of any of the SCFAs (Table 2), it was striking that, with the exception of a single correlation between OTU14 and butyrate, there was no further overlap in the OTU-SCFA correlation networks between the Control and PBLC treatments. Thus, differential network analysis for unique correlations in the Control group (Supplementary Figure S3A) largely mirrored the set of the all identified correlations in that group (Figure 6A), whereas differential network analysis for unique correlations in both PBLC groups (Supplementary Figure S3B) largely mirrored the total set of correlations identified in the PBLC groups (Figure 6B).

### Interactions Among Microbiota

**FIGURE 7:**
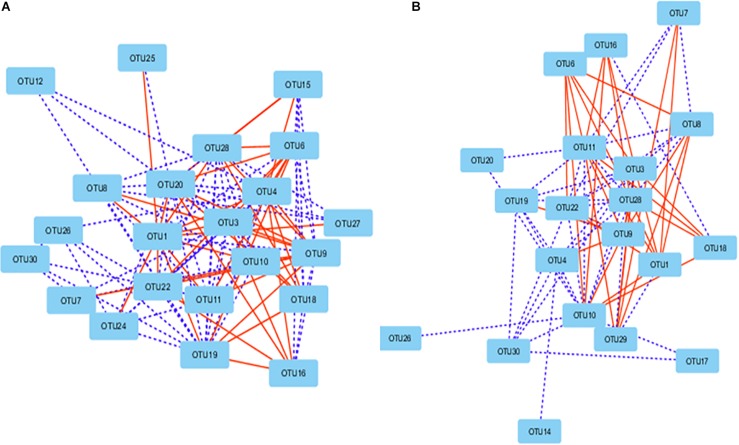
Correlation networks showing correlations between major OTUs (each representing ≥0.5% total sequences) in the rumen of the Control group **(A)** and both the plant bioactive lipid compound groups **(B)**. Dotted lines indicate positive correlations, while solid lines represent negative correlations. The shorter the distance between two nodes, the stronger the correlation between them. See Supplementary Table S5 for the taxonomic assignment of the OTUs.

Network analysis revealed that some ruminal microbes extensively correlated among themselves. A total of 106 correlations were found among the 22 OTUs in the correlation network of the Control group (*P* < 0.01; *r* > 0.62 or *r* <−0.62) either positively (*n* = 64) or negatively (*n* = 42) (Figure 7A). In the PBLC groups, 20 OTUs were correlated (*P* < 0.001; *r* > 0.62 or *r* < −0.62) to other OTUs forming 74 correlations, out of which 34 were negative (Figure 7B). The OTU-OTU correlations between the microbiota of the solid vs. liquid fractions were less prominent (Supplementary Figure S4). Differential network analysis revealed that 62 correlations (mostly positive correlations) among the 22 OTUs were exclusively present in the Control group compared with the PBLC groups (Supplementary Figure S5A). Alternatively, 19 OTUs in the PBLC groups were involved in 30 unique correlations (17 positive and 13 negative correlations) that were not present in the Control group (Supplementary Figure S5B). A merged network depicting all the OTU-OTU and the OTU-SCFA correlations is shown in Supplementary Figure S6 for completeness.

### Predicted Functions and Metabolic Pathways of the Bacterial Microbiota

At the KEGG level 1, all the functional categories had very small but significant differences (*P* ≤ 0.05) between the solid and the liquid fractions of the ruminal digesta (Supplementary Figure S7). The predicted genes for “metabolism” had the highest relative abundance (>52% of total functions in each fraction), which was significantly greater in the liquid than the solid fractions. The predicted “genetic information processing” was also more predominant for the liquid (24.1%) than for the solid (23.8%) fractions. However, “environmental information processing” (10.9% vs. 8.92%) and “cellular processes” (3.09% vs. 2.49%) were more predominant in the solid than in the liquid fractions. No significant difference in the predicted functional categories was noted at KEGG level 1 between the Control and the PBLC treatments (*P* > 0.05).

**FIGURE 8:**
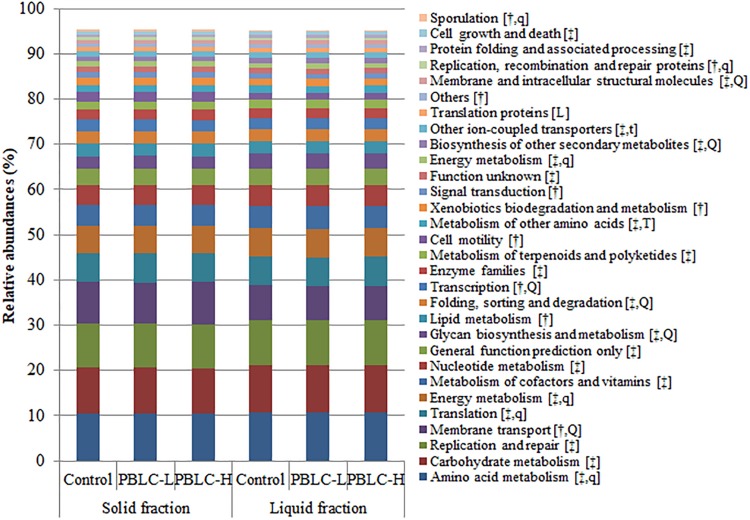
Relative abundances of different predicted functional features (at KEGG level 2) of the rumen microbiota compared between the solid and the liquid fractions of the ruminal digesta and among the three treatment groups. Control, PBLC-L, and PBLC-H, treatment groups supplemented with menthol-rich PBLC at 0, 80, and 160 mg/d, respectively. In the square brackets, symbols † and ‡ indicate grater (*P* ≤ 0.05) abundances in the solid and the liquid fractions, respectively, while uppercase letters indicate significant (*P* ≤ 0.05) treatment effects (T; Control vs. both PBLC-L and PBLC-H) or dose effect (L for linear, Q for quadratic) of PBLC; whereas, lowercase letters (t for treatment, and l and q for dose) indicate a trend (0.05 < *P* ≤ 0.10).

At KEGG level 2, a total of 64 KEGG ortholog groups were predicted. Among them, 30 gene families had a relative abundance >0.5%, and all of them were different between the solid and liquid fractions (*P* ≤ 0.05), except for the genes assigned to “translation proteins” (Figure 8 and Supplementary Table S4). The majority of the functions were involved in (for solid vs. liquid fractions) “amino acid metabolism” (10.4% vs. 10.8%), “carbohydrate metabolism” (10.1% vs. 10.3%), “replication and repair” (9.74% vs. 10.0%), “membrane transport” (9.28% vs. 7.54%), “translation” (6.30% vs. 6.47%), and “energy metabolism” (6.17% vs. 6.33%). Among other predicted functional categories each with a relative abundance >1%, “signal transduction,” “xenobiotics biodegradation and metabolism,” “cell motility,” “transcription,” and “lipid metabolism” were more prevalent in the solid than in the liquid fractions. The PBLC treatments altered a number of functions (*P* < 0.05), including “membrane transport” (quadratic effect; lowest in PBLC-L), “glycan biosynthesis and metabolism” (quadratic effect; highest in PBLC-L), “folding, sorting and degradation” (quadratic effect; highest in PBLC-L), “transcription” (quadratic effect; lowest in PBLC-L), “metabolism of other amino acids” (increased in PBLC groups), “biosynthesis of other secondary metabolites” (quadratic effect; highest in PBLC-L), “translation proteins” (decreased linearly), and “membrane and intracellular structural molecules” (quadratic effect; highest in PBLC-L).

**FIGURE 9:**
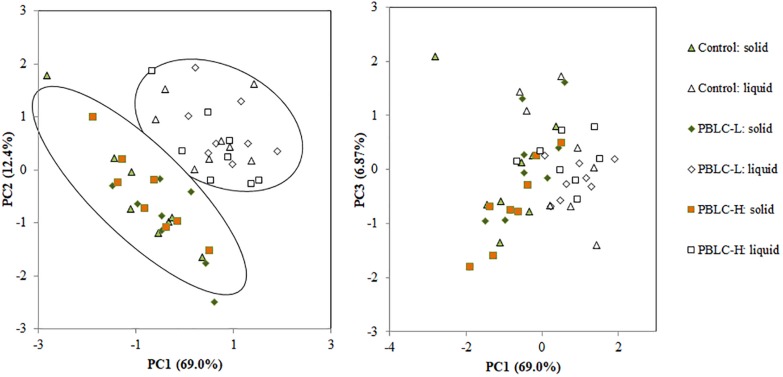
Principal component analysis of the predicted functional features (at KEGG level 3) of the ruminal microbiota between the solid and the liquid fractions of the ruminal digesta and among the three treatment groups. Control, PBLC-L, and PBLC-H, treatment groups supplemented with menthol-rich PBLC at 0, 80, and 160 mg/d, respectively. Statistical comparison of the scores of the first three principal components using MANOVA showed that solid vs. liquid fractions of digesta were different (*P* < 0.001). Treatment (*P* = 0.55) and interaction among treatment × digesta fraction (*P* = 0.47) were not significant for the overall predicted functional features.

At KEGG level 3, a total of 70 predicted functional categories each with a relative abundance >0.5% were noted in the samples (data not shown), and all these categories were different (*P* ≤ 0.05) in relative abundance between the two digesta fractions, with the exception of “ribosome biogenesis,” “glycolysis/gluconeogenesis,” “translation proteins_unclassified,” and “valine, leucine and isoleucine biosynthesis.” As evaluated using MANOVA analysis, the PCA plot also showed clear separation (Pillai’s trace, *P* < 0.001) between the two fractions (Figure 9). The PBLC treatments affected the relative abundance of “transporters” (quadratic effect; lowest in PBLC-L), “amino acid related enzymes” (quadratic effect; lowest in Control), “amino sugar and nucleotide sugar metabolism” (increased in PBLC groups), “transcription factors” (quadratic effect; lowest in PBLC-L), “chaperones and folding catalysts” (quadratic effect; highest in PBLC-L), “nitrogen metabolism” (increased in the PBLC groups), “membrane and intracellular structural molecules_unclassified” (quadratic effect; highest in PBLC-L), “protein export” (quadratic effect; lowest in Control), and “butanoate metabolism” (quadratic effect; lowest in PBLC-L), but the magnitudes of the changes were very small (Table 4). MANOVA analysis revealed that no separation was noted between the Control and the PBLC treatments (Pillai’s trace, *P* = 0.55) on the PCA plot (Figure 9). Furthermore, no significant interaction between the treatment × digesta fractions (Pillai’s trace, *P* = 0.47) was noted for the overall predicted functional features.

**TABLE 4:** Effect of supplementation of menthol-rich plant bioactive compounds (PBLC) on predicted gene function at KEGG level 3 in the solid and the liquid fractions of ruminal digesta in sheep.

**Categories^a^**	**Solid fraction^b^**			**Liquid fraction^b^**			**SEM**	***P*-value**	
				
	**Control**	**PBLC-L**	**PBLC-H**	**Control**	**PBLC-L**	**PBLC-H**		**Treatment**	**Fraction**
Transporters [†,Q]^c^	5.00	4.88	4.98	3.96	3.77	3.98	0.088	0.045	<0.001
Amino acid related enzymes [‡,Q]	1.59	1.60	1.60	1.64	1.65	1.65	0.005	0.040	<0.001
Amino sugar and nucleotide sugar metabolism [‡,T]	1.39	1.41	1.40	1.45	1.46	1.45	0.005	0.011	<0.001
Methane metabolism [‡,I]	1.34	1.35	1.30	1.26	1.23	1.27	0.003	0.073	<0.001
Transcription factors [†,Q]	1.34	1.28	1.31	1.08	1.03	1.08	0.028	0.049	<0.001
Chaperones and folding catalysts [‡,Q]	1.10	1.11	1.10	1.19	1.21	1.19	0.009	0.047	<0.001
Nitrogen metabolism [‡,T]	0.72	0.73	0.73	0.73	0.74	0.74	0.005	0.042	<0.001
Membrane and intracellular structural molecules_unclassified [‡,Q]	0.66	0.69	0.68	0.86	0.89	0.85	0.017	0.049	<0.001
Protein export [‡,Q]	0.67	0.68	0.68	0.71	0.71	0.71	0.003	0.033	<0.001
Butanoate metabolism [†,Q]	0.63	0.63	0.63	0.59	0.58	0.59	0.003	0.016	<0.001

**^*a*^Because a large number of gene categories were predicted at KEGG level 3, only the categories that both had relative gene abundances ≥0.5% and were significantly affected by PBLC were analyzed to find out major treatment effect. ^*b*^Control, PBLC-L, and PBLC-H, treatment groups supplemented with menthol-rich PBLC at 0, 80, and 160 mg/d, respectively. ^*c*^In the square brackets, symbols † and ‡ indicate greater (P ≤ 0.05) abundances in the solid and the liquid fractions, respectively, while uppercase letters indicate significant (P ≤ 0.05) treatment effect (T; Control vs. both PBLC-L and PBLC-H) or dose effect (L for linear, Q for quadratic) of PBLC; whereas, lowercase letters (t for treatment, and l and q for dose) indicate a trend (0.05 < P ≤ 0.10).**

## Discussion

### Effect of PBLC on Ruminal Fermentation

Various PBLC can influence the ruminal microbiota with consequences for fermentation, which may include an alteration of SCFA profile, inhibition of methane production, suppression of protein degradation and ammonia production, modification of fatty acid biohydrogenation, and mitigation of ruminal acidosis (Hutton et al., 2009; Patra, 2011; Cobellis et al., 2016). High doses of peppermint oil that contains menthol as a major component also changed ruminal SCFA profile (i.e., increased acetate and lowered propionate molar proportions), methane production and microbial abundances *in vitro* (Agarwal et al., 2009; Patra and Yu, 2012). However, the results from *in vitro* studies may not agree with those of *in vivo* studies (Flachowsky and Lebzien, 2012). Thus, we investigated how menthol-containing PBLC might affect ruminal microbiota and fermentation *in vivo*. The PBLC did not affect SCFA and ammonia concentrations or estimated methane production at the two doses tested. Our results are in line with a previous study that showed no effect of feeding peppermint herb (50 g/kg DM) to lactating Holstein cows on total SCFA concentration or molar proportion of acetate, propionate, and butyrate though digestibility of nutrients was lowered (Hosoda et al., 2005). Ando et al. (2003) also found no changes in ruminal SCFA concentration or molar SCFA proportion in steers supplemented with 200 g dried peppermint herb (equivalent to 29 g/kg diet). Assuming a menthol content of the applied peppermint supplements of ∼1% (Beigi et al., 2018), the PBLC doses used in those two previous studies were likely ∼5 times higher than the two doses used in our study. Hence, menthol does not appear to markedly change ruminal SCFA production when applied at dosages that have any practical relevance *in vivo*.

### Effect of PBLC on Ruminal Microbiota

According to previous studies (Larue et al., 2005; Pitta et al., 2010), the solid and the liquid fractions of rumen digesta have different microbiota. This can be explained by the fact that types and availability of substrates differ between the solid and the liquid fractions of the ruminal content, causing differences in the microbiota composition between these two fractions (de Menezes et al., 2011; Petri et al., 2012; Klevenhusen et al., 2017).

Supplementation of PBLC increased the microbial phylogenetic diversity and the number of observed species-level OTUs numerically (*P* = 0.14) in the solid fraction. This seems surprising since most studies revealed inhibitory or no effects of different PBLC on ruminal microbiota (Patra and Yu, 2012, 2015a; Schären et al., 2017, 2018). The inhibitory effect was also noted for menthol-containing peppermint oil that decreased the abundances of total bacteria, archaea, and select bacterial species including *Ruminococcus albus, Ruminococcus flavefaciens*, and *Fibrobacter succinogenes* in ruminal cultures *in vitro* (Patra and Yu, 2012). In agreement with the present study, however, peppermint oil increased species richness in a previous *in vitro* study, while garlic oil decreased richness when added at the same dose (0.5 g/L) (Patra and Yu, 2015b). Collectively, menthol at low or medium doses may increase diversity in the rumen by inhibiting some dominant bacteria.

The PBLC treatments altered the relative abundance of only a few taxa, and most of them were unclassified genera or species of bacteria. Among the classified genera, *Paludibacte*r and *BF311* were increased or increased linearly by PBLC. *Paludibacter* (e.g., *P. propionicigenes*) can ferment soluble sugars, soluble starch, and glycogen to predominantly propionate and acetate, but cannot utilize cellulose, xylan, fumarate, malate, lactate, succinate or pyruvate (Ueki et al., 2006). Little is known about the substrates and fermentation products of *BF311*, which has no cultured representative, but our correlation analysis suggests that this genus might interact with *Prevotella*, a major genus in the rumen (Bi et al., 2018).

Our initial hypothesis was that PBLC could have differential effects on the microbiota in the solid vs. the liquid fractions because those fractions have different microbiota (Belanche et al., 2017; Klevenhusen et al., 2017). However, we did not find much differential effect of PBLC between the two fractions except for *YRC22* and *Butyrivibrio*, with the former increasing its relative abundance in the liquid fraction while the latter expanding its relative abundance in the solid fraction with the increased doses of PBLC. This is different to our previous study *in vitro* (Patra and Yu, 2014) where *Butyrivibrio fibrisolvens*, a Gram-positive butyrate-producing species, was unaffected by peppermint oil but inhibited by oregano oil at the same dose. The discrepancy may be explained either by not having separate data for solid and liquid fractions or by the much higher dose of menthol-rich PBLC in the previous study performed *in vitro*. The metabolism of *YRC22* is not known because there is no known cultured representative, but it was predominant in the rumen of cattle (Klevenhusen et al., 2017) and increased due to dietary propionate supplementation (Yao et al., 2017) and decreased due to 1% nitrate addition (Zhao et al., 2015). Taken together, PBLC affected the abundance of certain ruminal bacteria, but these changes were rather small and were only marginally fraction-specific.

Ruminal archaeal taxa, i.e., *Euryarchaeota* and US_*Methanosphaera* in the solid fraction, *Thermoplasmata* and its family *Methanomassiliicoccaceae* and genus *vadinCA11* in the liquid fraction, were significantly decreased in the PBLC groups. *Thermoplasmata* is a class of methylotrophic methanogens, and it was also inhibited by rapeseed oil in lactating cows (Poulsen et al., 2013). These changes in archaea might suggest decreased methanogenesis by these taxa. Peppermint oil decreased methane production and total archaeal number in the ruminal fluid when used at very high concentrations *in vitro* (Agarwal et al., 2009; Patra and Yu, 2012) and acted similarly at lower doses *in vivo* in lactating dairy cattle (50 g/kg peppermint herbs; Hosoda et al., 2005). The relative proportions of individual SCFA have been shown to vary when methane production is altered (Patra, 2010), which has been used to estimate methane production from SCFA profiles in some studies (Wolin, 1960; Moss et al., 2000). The methane production estimated from the SCFA concentrations, however, did not change in response to the PBLC treatments. Albeit those estimates have to be interpreted with great care, they may suggest that changes in archaea do not necessarily cause an alteration in methane production, possibly due to compensation by the other methanogenic archaea (Ohene-Adjei et al., 2008).

### Correlations Between SCFA and Ruminal Microbiota

A correlation network existed between SCFA and some of the detected microbial taxa. Propionate and acetate showed correlations with one (i.e., US_*RF39*) and three (US_*Mogibacteriaceae*, US_*RF39*, and *Anaerostipes* sp.) OTUs, respectively, in the solid fraction, whereas those acids were not correlated to any OTUs in the liquid fraction. This might imply that acetate and propionate production is highly redundant in the ruminal microbiota. In fact, acetate and propionate concentrations in ruminal fluid are higher compared to the other SCFA, and many diverse ruminal microbes are involved in the production of these SCFA in their energy yielding pathways (Russell and Wallace, 1997). Butyrate had the most complex correlation network in both the solid- and liquid-associated microbiota. Moreover, three OTUs (US_*Ruminococcaceae* 1, *Ruminococcus* sp., and US_*Clostridiales* 2) all showed negative correlations to butyrate concentration in both the solid and the liquid fractions. This suggests that a rather limited number of microbes may be involved in the regulation of the production of butyrate (Diez-Gonzalez et al., 1999). Furthermore, the more complex networks in the solid fraction may imply that microbiota therein may be of greater importance for SCFA production in general and butyrate production in particular (Petri et al., 2012; Klevenhusen et al., 2017).

One striking finding of the present study was that the correlation networks between ruminal microbial OTUs and SCFA had almost no overlap between the Control group and the two PBLC-treated groups. Whereas propionate correlated with four microbial OTUs (i.e., US*_BS11*, US_*Prevotellaceae*, US_*Paraprevotellaceae*, and *Pseudobutyrivibrio* sp.) in the Control group, it was not correlated to any microbial taxa in the PBLC groups. Relationships between the other five measured SCFA and microbial taxa were also rather sparse in the PBLC groups compared to the Control. Importantly, the negative correlation between OTU14 (*Ruminococcus* spp.) and butyrate was the only correlation that occurred in both the Control and the PBLC groups. All other correlations were different between the Control and the PBLC treatments. This indicates that PBLC profoundly altered the correlation networks among OTUs and SCFA even though the relative abundance of only a few microbes was altered by PBLC and SCFA concentrations were unchanged. Previous studies had shown that diet could alter the metabolic networks in the rumen (Tapio et al., 2017; Wolff et al., 2017), however, we are not aware of any study that showed such effect on the correlation networks among OTUs and SCFA induced by PBLC.

### Interactions Among Microbiota

We also used co-occurrence networks to evaluate how PBLC might influence interactions among different taxa of ruminal microbes. We noted that most of the predominant taxa extensively interacted in both the Control and the PBLC groups. There were many unique interactions in the PBLC groups, which were absent in the Control group and vice versa. Thus, the PBLC treatment might extensively affect the microbial interactions despite the minor effects on rumen microbiota composition and relative abundance of major taxa and no detectable changes in SCFA production. The mechanistic explanations of these interactions are not known and neither it is known how they are affected by PBLC. The influence of diet on microbial interactions is at a similarly descriptive state (Henderson et al., 2015; Tapio et al., 2017). Thus, further research is warranted to better understand the physiology of the ruminal microbiota using metagenomics and metatranscriptomics in combination with fermentation profile analyses (Tapio et al., 2017).

### Functional Redundancy in the Ruminal Microbiota

As expected, most of the predicted functions differed between the solid- and the liquid-associated microbiota, which was further confirmed by PCA that separated solid and liquid fractions by the first two principal components. The differential functions between the two fractions were consistent with the finding of the study of Mao et al. (2015). Overall, although a few functional categories were affected by PBLC, no distinct separation among the treatments was seen on the PCA plot, suggesting that PBLC had little effect on the metabolic function of the microbiota. This once again highlights the functional redundancy of the ecosystem where the microbiota can adapt to fulfill its metabolic functions under varying environmental conditions.

## Conclusion

Menthol-rich PBLC at practically relevant dosages (80–160 mg/d; equivalent to 57–114 mg/kg diet DM) elicits a few distinct changes in microbiota composition, including a reduction of certain methanogens and an increase in microbiota diversity, especially among the particle-colonizing bacteria. However, the menthol-rich PBLC did not change ruminal microbial fermentation. Considering the changes in microbial composition and diversity, it appears surprising that PBLC rich in menthol did not exert a significant effect on ruminal fermentation. By demonstrating changes in the microbial and metabolic networks during PBLC supplementation, the present study indicates that stable fermentation is maintained during minor microbial alterations as a result of metabolic redundancy of the ruminal ecosystem. These findings suggest that correlation network analysis can add novel dimensions to the understanding of ruminal microbial interactions, which may be of practical relevance for targeted manipulation of ruminal fermentation. Further research is needed to elucidate and confirm these interactions.

## Data Availability

The datasets generated for this study can be found in NCBI Sequence Read Archive (SRA), PRJNA529255.

## Ethics Statement

The study was performed with ethical approval by the local authorities, the Landesamt für Gesundheit und Soziales, Berlin, Germany (LAGeSo, filed under G0141/17).

## Author Contributions

AP, SG, H-SB, and JA designed and conducted the experiment. AP and RP designed the feeding. TP, RP, and JA took responsibility for the microbiota, SCFA and ammonia analyses, respectively. AP analyzed all final data sets and created tables and figures. AP wrote the manuscript together with JA, ZY, and TP. H-SB, AP, and JA obtained funding for this study. All authors reviewed, provided comments and approved the final version of the manuscript.

## Conflict of Interest Statement

H-SB is a member of PerformaNat GmbH, Germany, which funded parts of the study. This fact had no influence on the acquisition and interpretation of results and does not affect authors’ adherence to the publishing policies of Frontiers. The remaining authors declare that the research was conducted in the absence of any commercial or financial relationships that could be construed as a potential conflict of interest.

## Footnotes

1 https://mcic.osu.edu/home

2 http://huttenhower.sph.harvard.edu/galaxy
