# Supplementary material for: Dietary Bioactive Lipid Compounds Rich in Menthol Alter Interactions Among Members of Ruminal Microbiota in Sheep
Source: Front Microbiol. 2019 Sep 4;10:2038. doi: 10.3389/fmicb.2019.02038 (PMC6738200; doi:10.3389/fmicb.2019.02038)
Supplement: Supplementary file 1 [file Table_1.doc]

**TABLE S1 |** Relative abundances of bacterial and archaeal phyla in the solid and the liquid fractions of ruminal digesta of different dietary groups.

| **Phylum** | **Solid fraction^a^** | | | **Liquid fraction^a^** | | | **SEM** | ***P*-value** | |
| --- | --- | --- | --- | --- | --- | --- | --- | --- | --- |
|  | **Control** | **PBLC-L** | **PBLC-H** | **Control** | **PBLC-L** | **PBLC-H** |  | **Treatment** | **Fraction** |
| *Bacteroidetes* [‡]^b^ | 53.7 | 56.3 | 55.0 | 70.7 | 73.6 | 71.4 | 1.56 | 0.10 | <0.001 |
| *Firmicutes* [†,q] | 40.9 | 38.3 | 40.1 | 22.8 | 19.8 | 22.1 | 1.49 | 0.065 | <0.001 |
| *Fibrobacteres* [†] | 1.60 | 1.48 | 1.26 | 0.16 | 0.30 | 0.12 | 0.070 | 0.25 | <0.001 |
| *Spirochaetes* [†] | 0.91 | 0.79 | 0.77 | 0.56 | 0.49 | 0.50 | 0.12 | 0.34 | 0.004 |
| *Tenericutes* | 0.54 | 0.58 | 0.59 | 0.56 | 0.47 | 0.67 | 0.090 | 0.38 | 0.99 |
| *Verrucomicrobia* [‡] | 0.53 | 0.53 | 0.55 | 1.01 | 0.98 | 1.21 | 0.078 | 0.59 | 0.001 |
| *Chloroflexi* [Q] | 0.32 | 0.60 | 0.27 | 0.30 | 0.44 | 0.19 | 0.094 | 0.004 | 0.27 |
| *Euryarchaeota* [T] | 0.40 | 0.15 | 0.11 | 0.34 | 0.13 | 0.36 | 0.054 | 0.009 | 0.26 |
| *Proteobacteria* [‡] | 0.29 | 0.20 | 0.40 | 0.46 | 0.38 | 0.37 | 0.073 | 0.18 | 0.078 |
| *Synergistetes* [‡] | 0.14 | 0.14 | 0.23 | 2.02 | 2.39 | 2.00 | 0.11 | 0.66 | <0.001 |
| *TM7* | 0.11 | 0.17 | 0.18 | 0.24 | 0.14 | 0.19 | 0.034 | 0.40 | 0.23 |
| *SR1* [†,l] | 0.13 | 0.09 | 0.22 | 0.021 | 0.033 | 0.048 | 0.028 | 0.051 | <0.001 |
| *Planctomycetes* [†,t] | 0.10 | 0.085 | 0.074 | 0.045 | 0.016 | 0.018 | 0.015 | 0.062 | <0.001 |
| *Elusimicrobia* [†] | 0.093 | 0.082 | 0.072 | 0.035 | 0.059 | 0.045 | 0.023 | 0.66* | 0.024* |
| *Actinobacteria* [†] | 0.074 | 0.071 | 0.064 | 0.020 | 0.007 | 0.025 | 0.011 | 0.75* | <0.001* |
| *Cyanobacteria* [‡] | 0.042 | 0.046 | 0.036 | 0.15 | 0.19 | 0.13 | 0.023 | 0.25 | <0.001 |
| UP_Bacteria [‡,Q] | 0.035 | 0.024 | 0.042 | 0.12 | 0.043 | 0.12 | 0.027 | 0.007 | 0.003 |
| *Lentisphaerae* [‡] | 0.022 | 0.031 | 0.037 | 0.42 | 0.47 | 0.52 | 0.074 | 0.42 | <0.001 |
| *WPS-2* | 0.022 | 0.041 | 0.005 | 0.016 | 0.025 | 0.008 | 0.013 | 0.76* | 0.56* |
| *LD1* [‡]] | 0.015 | 0.011 | 0.014 | 0.050 | 0.041 | 0.047 | 0.008 | 0.44 | <0.001 |
| *Armatimonadetes* | 0.000 | 0.001 | 0.002 | 0.006 | 0.0 | 0.005 | 0.003 | 0.84* | 0.85* |

^a^Control, PBLC-L, and PBLC-H, dietary treatment groups supplemented with menthol-rich PBLC at 0, 80 and 160 mg/d, respectively.

^b^In the square brackets, symbols † and ‡ indicate greater (*P* ≤ 0.05) abundances in the solid and the liquid fractions, respectively, while uppercase letters indicate significant (*P* ≤ 0.05) treatment effect (T; Control vs. PBLC-L and PBLC-H) or dose effect (L for linear, Q for quadratic) of PBLC; whereas, lowercase letters (t for treatment, l for linear, q for quadratic dose effect) indicate a trend (0.05 < *P* ≤ 0.10).

*Wilcoxon test was used because residuals did not follow normality.

No interactions between treatment and digesta fraction were present (*P* ≥ 0.10).
