# Supplementary material for: Dietary Bioactive Lipid Compounds Rich in Menthol Alter Interactions Among Members of Ruminal Microbiota in Sheep
Source: Front Microbiol. 2019 Sep 4;10:2038. doi: 10.3389/fmicb.2019.02038 (PMC6738200; doi:10.3389/fmicb.2019.02038)
Supplement: Supplementary file 2 [file Table_2.doc]

**TABLE S2 |** Relative abundances of major bacterial families (each representing ≥ 0.5% total sequences in at least one solid or one liquid fraction sample) in the solid and the liquid fractions of ruminal digesta of different dietary groups.

| **Family** | **Solid fraction^a^** | | | **Liquid fraction^a^** | | | **SEM** | ***P*-value** | |
| --- | --- | --- | --- | --- | --- | --- | --- | --- | --- |
|  | **Control** | **PBLC-L** | **PBLC-H** | **Control** | **PBLC-L** | **PBLC-H** |  | **Treatment** | **Fraction** |
| *Prevotellaceae* [‡]^b^ | 21.7 | 22.2 | 24.5 | 38.1 | 40.7 | 36.9 | 1.37 | 0.52 | <0.001 |
| UF_*Clostridiales* 1[†] | 12.9 | 12.4 | 11.9 | 5.81 | 4.66 | 5.12 | 0.57 | 0.25 | <0.001 |
| *Ruminococcaceae* [†] | 12.7 | 11.0 | 11.4 | 7.27 | 5.64 | 6.54 | 0.95 | 0.22 | <0.001 |
| UF_*Bacteroidales* 1[‡] | 11.3 | 9.32 | 10.6 | 13.8 | 13.2 | 12.3 | 1.01 | 0.40 | 0.002 |
| *Lachnospiraceae* [†,L] | 6.46 | 6.96 | 8.10 | 3.75 | 3.45 | 4.18 | 0.42 | 0.019 | <0.001 |
| *S24-7* [†] | 6.23 | 6.46 | 6.21 | 2.21 | 1.59 | 1.69 | 0.17 | 0.76 | <0.001 |
| *BS11* [Q] | 5.09 | 8.25 | 4.73 | 4.30 | 5.35 | 4.55 | 0.94 | 0.012 | 0.10 |
| UF_*Bacteroidales* 2[†] | 5.12 | 5.86 | 4.47 | 1.91 | 1.69 | 1.97 | 0.44 | 0.45 | <0.001 |
| *Paraprevotellaceae* [‡] | 3.21 | 3.07 | 2.99 | 3.91 | 3.84 | 4.92 | 0.40 | 0.43 | 0.001 |
| *Clostridiaceae* [†] | 2.86 | 2.64 | 2.54 | 0.64 | 0.60 | 0.54 | 0.073 | 0.43 | <0.001 |
| *Christensenellaceae* [†,T] | 1.88 | 1.23 | 1.46 | 0.67 | 0.30 | 0.52 | 0.092 | 0.010 | <0.001 |
| *Veillonellaceae* [‡] | 1.42 | 1.35 | 1.70 | 3.22 | 3.68 | 3.34 | 0.11 | 0.30 | <0.001 |
| *Fibrobacteraceae* [†] | 1.60 | 1.48 | 1.26 | 0.16 | 0.30 | 0.12 | 0.14 | 0.27 | <0.001 |
| UF_*Clostridiales* 2 [†,l] | 1.33 | 1.39 | 1.58 | 0.63 | 0.77 | 0.79 | 0.11 | 0.060 | <0.001 |
| *Mogibacteriaceae* [†] | 1.09 | 1.02 | 1.04 | 0.30 | 0.24 | 0.32 | 0.062 | 0.50 | <0.001 |
| *Spirochaetaceae* [†] | 0.89 | 0.77 | 0.75 | 0.45 | 0.43 | 0.42 | 0.11 | 0.71 | <0.001 |
| *Bacteroidaceae* [‡,L] | 0.65 | 0.71 | 0.89 | 0.73 | 1.15 | 1.18 | 0.15 | 0.026 | 0.030 |
| *RF16* [‡] | 0.37 | 0.37 | 0.53 | 5.33 | 5.76 | 6.69 | 0.59 | 0.42 | <0.001 |
| *Erysipelotrichaceae* [‡,l,i] | 0.27 | 0.26 | 0.26 | 0.51 | 0.48 | 0.70 | 0.052 | 0.090 | <0.001 |
| *RFP12* [‡] | 0.22 | 0.17 | 0.18 | 0.75 | 0.65 | 0.93 | 0.12 | 0.47 | <0.001 |
| *Dethiosulfovibrionaceae* [‡] | 0.14 | 0.14 | 0.23 | 2.02 | 2.39 | 2.00 | 0.11 | 0.65 | <0.001 |
| *Porphyromonadaceae* [‡,T] | 0.06 | 0.03 | 0.12 | 0.14 | 0.28 | 0.63 | 0.058 | 0.008* | 0.008* |

^a^Control, PBLC-L, and PBLC-H, dietary treatment groups fed menthol-rich plant bioactive lipid compounds at 0, 80 and 160 mg/d, respectively.

^b^In the square brackets, symbols † and ‡ indicate greater (*P* ≤ 0.05) abundances in the solid and the liquid fractions, respectively, while uppercase letters indicate significant (*P* ≤ 0.05) treatment effect (T; Control vs. PBLC-L and PBLC-H) or dose effect (L for linear, Q for quadratic) of PBLC; whereas, lowercase letters (t for treatment, l and q for linear and quadratic dose effect, respectively, and i for interaction effect between treatment and digesta fraction) indicate a trend (0.05 < *P* ≤ 0.10).

* Wilcoxon test was used because residuals did not follow normality.
