# Supplementary material for: Dietary Bioactive Lipid Compounds Rich in Menthol Alter Interactions Among Members of Ruminal Microbiota in Sheep
Source: Front Microbiol. 2019 Sep 4;10:2038. doi: 10.3389/fmicb.2019.02038 (PMC6738200; doi:10.3389/fmicb.2019.02038)
Supplement: Supplementary file 3 [file Table_3.doc]

**TABLE S3 |** Relative abundances of major genera (each representing ≥ 0.5% total sequences in at least one solid or one liquid fraction) in the solid and the liquid fractions of ruminal digesta of different dietary groups.

| **Genus** | **Solid fraction^a^** | | | **Liquid fraction^a^** | | | **SEM** | ***P*-value** | |
| --- | --- | --- | --- | --- | --- | --- | --- | --- | --- |
|  | **Control** | **PBLC-L** | **PBLC-H** | **Control** | **PBLC-L** | **PBLC-H** |  | **Treatment** | **Fraction** |
| *Prevotella* [‡]^b^ | 19.8 | 20.2 | 22.4 | 36.9 | 39.4 | 35.9 | 1.46 | 0.38 | <0.001 |
| UG_*Clostridiales* 1[†,t] | 12.9 | 12.4 | 11.9 | 5.81 | 4.66 | 5.12 | 0.57 | 0.098 | <0.001 |
| UG_*Bacteroidales* 1[‡] | 11.3 | 9.32 | 10.6 | 13.8 | 13.2 | 12.3 | 1.01 | 0.19 | 0.002 |
| UG_*Ruminococcaceae* 1[†] | 7.37 | 6.87 | 7.14 | 4.10 | 3.61 | 3.95 | 0.52 | 0.37 | <0.001 |
| UG_*S24-7* [†] | 6.23 | 6.46 | 6.21 | 2.21 | 1.59 | 1.69 | 0.17 | 0.69 | <0.001 |
| UG_*BS11* [Q] | 5.09 | 8.25 | 4.73 | 4.30 | 5.35 | 4.55 | 0.94 | 0.012 | 0.10 |
| UG_*Bacteroidales* 2[†] | 5.12 | 5.86 | 4.47 | 1.91 | 1.69 | 1.97 | 0.44 | 0.28 | <0.001 |
| UG_*Lachnospiraceae* 1[†,L] | 3.46 | 4.01 | 4.50 | 1.79 | 1.76 | 2.08 | 0.27 | 0.018 | <0.001 |
| *Ruminococcus* [†] | 4.04 | 3.06 | 3.04 | 2.02 | 1.33 | 1.75 | 0.56 | 0.14 | <0.001 |
| *Clostridium* [†] | 2.84 | 2.62 | 2.53 | 0.63 | 0.60 | 0.53 | 0.075 | 0.42 | <0.001 |
| UG_*Prevotellaceae* [†] | 1.90 | 2.00 | 2.08 | 1.20 | 1.33 | 1.04 | 0.29 | 0.87* | <0.001* |
| *YRC22* [L,i] | 1.48 | 1.62 | 1.56 | 1.04 | 1.25 | 2.17 | 0.25 | 0.022 | 0.76 |
| UG_*Christensenellaceae* [†,t] | 1.88 | 1.23 | 1.46 | 0.67 | 0.29 | 0.51 | 0.19 | 0.080* | <0.001* |
| *Fibrobacter* [†] | 1.60 | 1.48 | 1.25 | 0.16 | 0.30 | 0.12 | 0.075 | 0.25 | <0.001 |
| UG_*Clostridiales* 2[†,l] | 1.33 | 1.39 | 1.58 | 0.63 | 0.77 | 0.79 | 0.11 | 0.057 | <0.001 |
| *Butyrivibrio* [†,q,i] | 1.17 | 1.24 | 1.67 | 1.09 | 0.85 | 1.02 | 0.13 | 0.078 | <0.001 |
| *Succiniclasticum* [†] | 0.94 | 0.91 | 1.21 | 0.28 | 0.68 | 0.36 | 0.14 | 0.15 | <0.001 |
| *CF231* [‡] | 0.93 | 0.91 | 0.97 | 1.74 | 1.63 | 1.93 | 0.17 | 0.42 | <0.001 |
| UG_*Mogibacteriaceae* [†] | 0.94 | 0.87 | 0.92 | 0.24 | 0.18 | 0.26 | 0.051 | 0.14 | <0.001 |
| UG_*Ruminococcaceae* 2[†,Q] | 0.94 | 0.80 | 0.88 | 0.63 | 0.42 | 0.65 | 0.09 | 0.047 | <0.001 |
| *Treponema* [†] | 0.88 | 0.75 | 0.74 | 0.44 | 0.41 | 0.40 | 0.11 | 0.37 | <0.001 |
| *BF311* [‡,L] | 0.65 | 0.71 | 0.89 | 0.73 | 1.15 | 1.18 | 0.15 | 0.025 | 0.030 |
| UG_*Lachnospiraceae* 2[†,Q] | 0.75 | 0.59 | 0.77 | 0.43 | 0.36 | 0.54 | 0.082 | 0.050 | <0.001 |
| UG_*Paraprevotellaceae* [‡,L] | 0.74 | 0.52 | 0.44 | 1.03 | 0.89 | 0.79 | 0.13 | 0.036 | 0.002 |
| UG_*RF16* [‡] | 0.37 | 0.37 | 0.53 | 5.33 | 5.76 | 6.69 | 0.59 | 0.20 | <0.001 |
| UG_*Veillonellaceae* [‡] | 0.33 | 0.27 | 0.35 | 2.02 | 1.91 | 2.20 | 0.29 | 0.58 | <0.001 |
| UG_*RFP12* [‡] | 0.22 | 0.17 | 0.18 | 0.75 | 0.65 | 0.93 | 0.12 | 0.29 | <0.001 |
| *TG5* [‡] | 0.13 | 0.12 | 0.21 | 2.01 | 2.38 | 2.00 | 0.11 | 0.86 | <0.001 |
| *Paludibacter* [T] | 0.085 | 0.060 | 0.14 | 0.17 | 0.31 | 0.68 | 0.074 | 0.043* | 0.14* |

^a^Control, PBLC-L, and PBLC-H, dietary treatment groups supplemented with menthol-rich PBLC at 0, 80 and 160 mg/d, respectively.

^b^In the square brackets, symbols † and ‡ indicate greater (*P* ≤ 0.05) abundances in the solid and the liquid fractions, respectively, while uppercase letters indicate significant (*P* ≤ 0.05) treatment effect (T; Control vs. PBLC-L and PBLC-H) or dose effect (L for linear, Q for quadratic and I for interaction) of PBLC; whereas, lowercase letters (t for treatment, l and q for linear and quadratic dose effect, respectively, and i for interaction effect between treatment and digesta fraction) indicate a trend (0.05 < *P* ≤ 0.10).

* Wilcoxon test was used because residuals did not follow normality.
