# Supplementary material for: Dietary Bioactive Lipid Compounds Rich in Menthol Alter Interactions Among Members of Ruminal Microbiota in Sheep
Source: Front Microbiol. 2019 Sep 4;10:2038. doi: 10.3389/fmicb.2019.02038 (PMC6738200; doi:10.3389/fmicb.2019.02038)
Supplement: Supplementary file 4 [file Table_4.doc]

**TABLE S4 |** Effect of supplementation of menthol-rich plant bioactive compounds (PBLC) on predicted gene function at KEGG level 2 in the solid and the liquid fractions of ruminal digesta in sheep.

| **Categories^a^** | **Solid fractions^b^** | | | **Liquid fraction^b^** | | | **SEM** | ***P*-value** | |
| --- | --- | --- | --- | --- | --- | --- | --- | --- | --- |
|  | **Control** | **PBLC-L** | **PBLC-H** | **Control** | **PBLC-L** | **PBLC-H** |  | **Treatment** | **Fraction** |
| Amino acid metabolism [‡,q]^c^ | 10.40 | 10.43 | 10.39 | 10.74 | 10.81 | 10.73 | 0.032 | 0.055 | <0.001 |
| Carbohydrate metabolism [‡] | 10.15 | 10.11 | 10.10 | 10.35 | 10.32 | 10.34 | 0.048 | 0.86* | <0.001* |
| Replication and repair [‡] | 9.75 | 9.75 | 9.73 | 10.02 | 10.05 | 9.98 | 0.029 | 0.24 | <0.001 |
| Membrane transport [†,Q] | 9.35 | 9.16 | 9.34 | 7.63 | 7.32 | 7.67 | 0.140 | 0.049 | <0.001 |
| Translation [‡,q] | 6.30 | 6.32 | 6.27 | 6.46 | 6.48 | 6.46 | 0.017 | 0.076 | <0.001 |
| Energy metabolism [‡,q] | 6.12 | 6.23 | 6.16 | 6.30 | 6.35 | 6.33 | 0.041 | 0.087 | <0.001 |
| Metabolism of cofactors and vitamins [‡] | 4.57 | 4.62 | 4.61 | 4.87 | 4.94 | 4.88 | 0.033 | 0.10 | <0.001 |
| Nucleotide metabolism [‡] | 4.39 | 4.41 | 4.38 | 4.60 | 4.62 | 4.59 | 0.018 | 0.14 | <0.001 |
| General function prediction only [‡] | 3.58 | 3.59 | 3.58 | 3.60 | 3.60 | 3.61 | 0.007 | 0.33 | <0.001 |
| Glycan biosynthesis and metabolism [‡,Q] | 2.76 | 2.86 | 2.80 | 3.33 | 3.44 | 3.33 | 0.055 | 0.045 | <0.001 |
| Lipid metabolism [†] | 2.80 | 2.78 | 2.76 | 2.73 | 2.72 | 2.74 | 0.015 | 0.37 | <0.001 |
| Folding, sorting and degradation [‡,Q] | 2.63 | 2.65 | 2.64 | 2.75 | 2.78 | 2.75 | 0.014 | 0.035 | <0.001 |
| Transcription [†,Q] | 2.63 | 2.56 | 2.60 | 2.35 | 2.29 | 2.34 | 0.029 | 0.036 | <0.001 |
| Enzyme families [‡] | 2.25 | 2.25 | 2.25 | 2.27 | 2.27 | 2.26 | 0.006 | 0.25 | <0.001 |
| Metabolism of terpenoids and polyketides [‡] | 1.77 | 1.77 | 1.77 | 1.84 | 1.86 | 1.84 | 0.008 | 0.12 | <0.001 |
| Cell motility [†] | 2.13 | 2.06 | 2.18 | 1.46 | 1.36 | 1.46 | 0.069 | 0.11 | <0.001 |
| Metabolism of other amino acids [‡,T] | 1.52 | 1.55 | 1.55 | 1.64 | 1.68 | 1.65 | 0.013 | 0.041 | <0.001 |
| Xenobiotics biodegradation and metabolism [†] | 1.55 | 1.51 | 1.53 | 1.49 | 1.47 | 1.48 | 0.001 | 0.20 | <0.001 |
| Signal transduction [†] | 1.41 | 1.40 | 1.41 | 1.19 | 1.17 | 1.20 | 0.017 | 0.23 | <0.001 |
| Function unknown [‡] | 1.17 | 1.17 | 1.16 | 1.19 | 1.18 | 1.19 | 0.005 | 0.12 | <0.001 |
| Energy metabolism [‡,q] | 1.08 | 1.10 | 1.09 | 1.16 | 1.17 | 1.16 | 0.010 | 0.086 | <0.001 |
| Biosynthesis of other secondary metabolites [‡,Q] | 1.06 | 1.07 | 1.07 | 1.17 | 1.19 | 1.17 | 0.010 | 0.049 | <0.001 |
| Other ion-coupled transporters [‡,t] | 1.08 | 1.08 | 1.09 | 1.13 | 1.15 | 1.14 | 0.006 | 0.055 | <0.001 |
| Translation proteins [L] | 0.993 | 0.990 | 0.985 | 0.996 | 0.992 | 0.992 | 0.003 | 0.031 | 0.13 |
| Others [†] | 0.794 | 0.787 | 0.801 | 0.775 | 0.777 | 0.778 | 0.005 | 0.24 | <0.001 |
| Membrane and intracellular structural molecules [‡,Q] | 0.662 | 0.694 | 0.683 | 0.856 | 0.895 | 0.854 | 0.017 | 0.049 | <0.001 |
| Replication, recombination and repair proteins [†,q] | 0.733 | 0.720 | 0.724 | 0.654 | 0.639 | 0.652 | 0.007 | 0.086 | <0.001 |
| Protein folding and associated processing [‡] | 0.611 | 0.618 | 0.618 | 0.650 | 0.654 | 0.648 | 0.003 | 0.14 | <0.001 |
| Cell growth and death [‡] | 0.576 | 0.581 | 0.582 | 0.609 | 0.617 | 0.608 | 0.004 | 0.110 | <0.001 |
| Sporulation [†,q] | 0.644 | 0.588 | 0.605 | 0.369 | 0.314 | 0.361 | 0.028 | 0.077 | <0.001 |

^a^Because a large number of gene categories were predicted at KEGG level 2, only the categories with relative gene abundances ≥ 0.5% were analysed to find out significant differences.

^b^Control, PBLC-L, and PBLC-H, dietary treatment groups supplemented with menthol-rich PBLC at 0, 80 and 160 mg/d, respectively.

^c^In the square brackets, symbols † and ‡ indicate grater (*P* ≤ 0.05) abundances in the solid and the liquid fractions, respectively, while uppercase letters indicate significant (*P* ≤ 0.05) treatment effect (T; Control vs. PBLC-L and PBLC-H) or dose effect (L for linear, Q for quadratic) of PBLC; whereas, lowercase letters (t for treatment, and l and q for dose) indicate a trend (0.05 < *P* ≤ 0.10).

* Wilcoxon test was used because residuals did not follow normality.
