# Supplementary material for: Dietary Bioactive Lipid Compounds Rich in Menthol Alter Interactions Among Members of Ruminal Microbiota in Sheep
Source: Front Microbiol. 2019 Sep 4;10:2038. doi: 10.3389/fmicb.2019.02038 (PMC6738200; doi:10.3389/fmicb.2019.02038)
Supplement: Supplementary file 5 [file Table_5.DOC]

**TABLE S5 |** Operation taxonomic unit (OTUs) with relative abundances of ≥ 0.1% used in the analyses of correlated networks between microbiota and short-chain fatty acids.

| OTU1 | *Prevotella* sp. 1 | OTU27 | US*_Lachnospiraceae* 2 |
| --- | --- | --- | --- |
| OTU2 | US*_Bacteroidales* 1 | OTU28 | US_*Mogibacteriaceae* |
| OTU3 | US*_Clostridiales* 1 | OTU29 | *Prevotella* sp. 2 |
| OTU4 | US*_Ruminococcaceae* 1 | OTU30 | *Ruminococcus flavefaciens* |
| OTU5 | US*_BS11* | OTU31 | US*_RFP12* |
| OTU6 | *Prevotella ruminicola* | OTU32 | *SHD-231* sp. |
| OTU7 | US*_S24-7* | OTU33 | *Paludibacter* sp*.* |
| OTU8 | US*_Bacteroidales* 2 | OTU34 | US_*RF39* |
| OTU9 | US*_RF16* | OTU35 | *Ruminococcus bromii* |
| OTU10 | US*_Lachnospiraceae* 1 | OTU36 | *Coprococcus* sp. |
| OTU11 | *Clostridium* sp. | OTU37 | *Methanobrevibacter* sp. |
| OTU12 | US_*Prevotellaceae* | OTU38 | *Oscillospira* sp*.* |
| OTU13 | *YRC22* sp. | OTU39 | *Selenomonas ruminantium* |
| OTU14 | *Ruminococcus* sp. 1 | OTU40 | *RFN20* sp*.* |
| OTU15 | *CF231* sp. | OTU41 | US_*Veillonellaceae* 2 |
| OTU16 | US_*Veillonellaceae* 1 | OTU42 | US_*Victivallaceae* 3 |
| OTU17 | *Butyrivibrio* sp. | OTU43 | US_*R4-41B* |
| OTU18 | *TG5* sp. | OTU44 | *Anaeroplasma* sp. |
| OTU19 | US_*Clostridiales* 2 | OTU45 | *Pseudobutyrivibrio* sp. |
| OTU20 | US*_Christensenellaceae* | OTU46 | US_*F16* |
| OTU21 | *BF311* sp. | OTU47 | *Desulfovibrio* sp. |
| OTU22 | *Fibrobacter succinogenes* | OTU48 | *Ruminococcus* sp. 2 |
| OTU23 | US_*Paraprevotellaceae* | OTU49 | *Moryella* sp. |
| OTU24 | *Succiniclasticum* sp. | OTU50 | *Mogibacterium* sp. |
| OTU25 | US*_Ruminococcaceae* 2 | OTU51 | *Anaerostipes* sp. |
| OTU26 | *Treponema* sp. |  |  |

OTUs were clustered based on 99% sequence similarity. US = unclassified species.
