# Supplementary figures and images for: Dietary Bioactive Lipid Compounds Rich in Menthol Alter Interactions Among Members of Ruminal Microbiota in Sheep
Source: Front Microbiol. 2019 Sep 4;10:2038. doi: 10.3389/fmicb.2019.02038 (PMC6738200; doi:10.3389/fmicb.2019.02038)

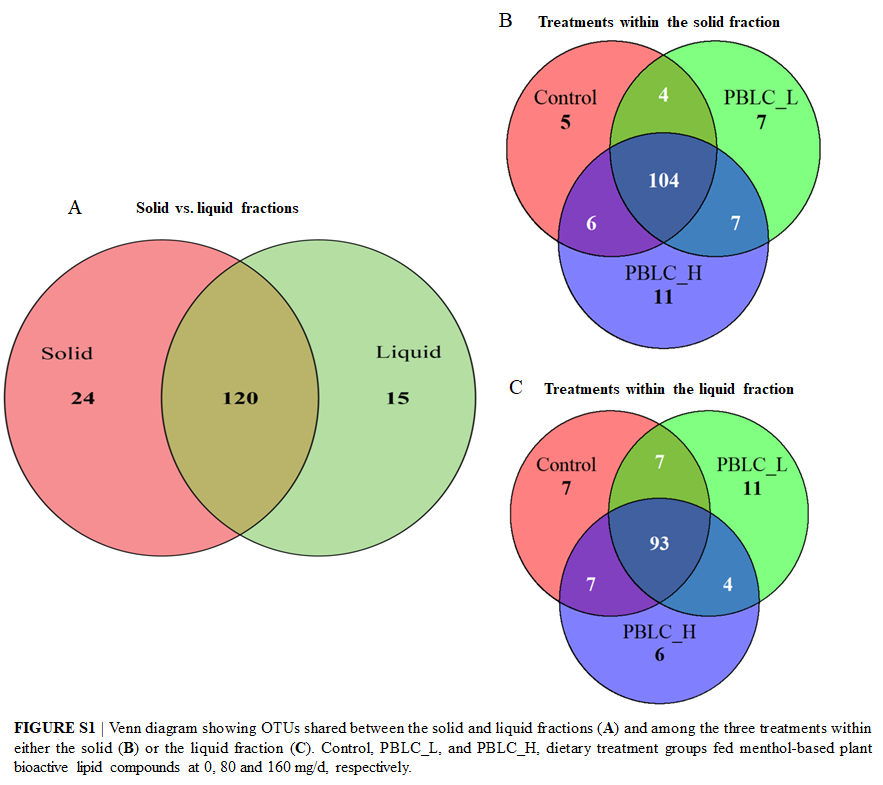

Supplement: Supplementary file 6 [file Image_1.TIF]

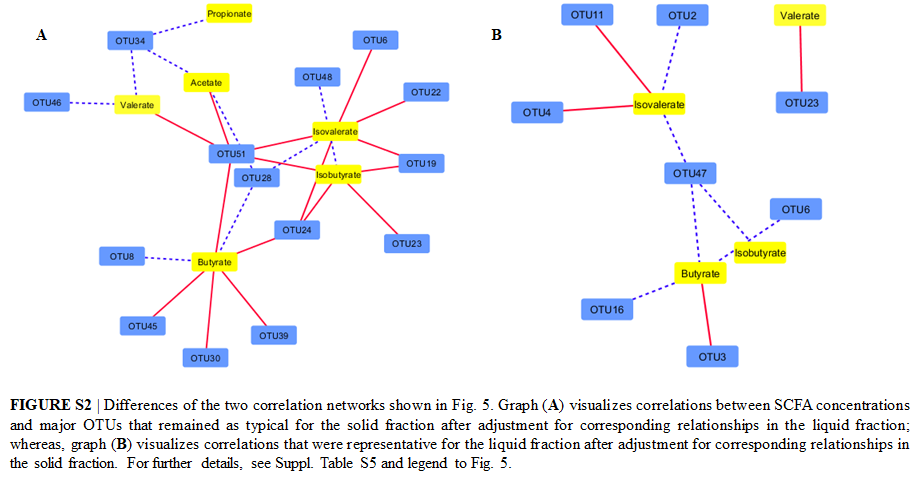

Supplement: Supplementary file 7 [file Image_2.TIF]

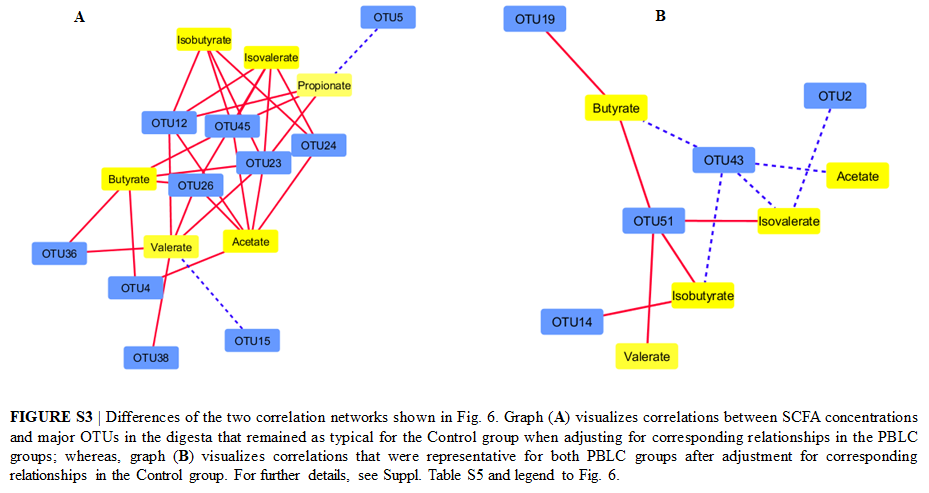

Supplement: Supplementary file 8 [file Image_3.TIF]

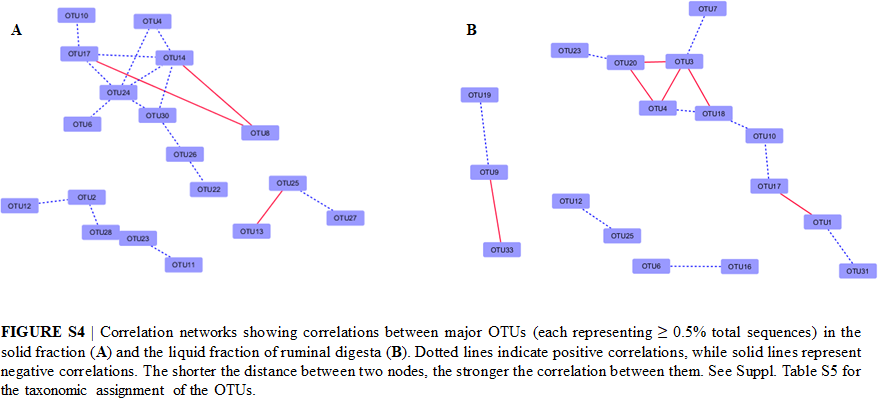

Supplement: Supplementary file 9 [file Image_4.TIF]

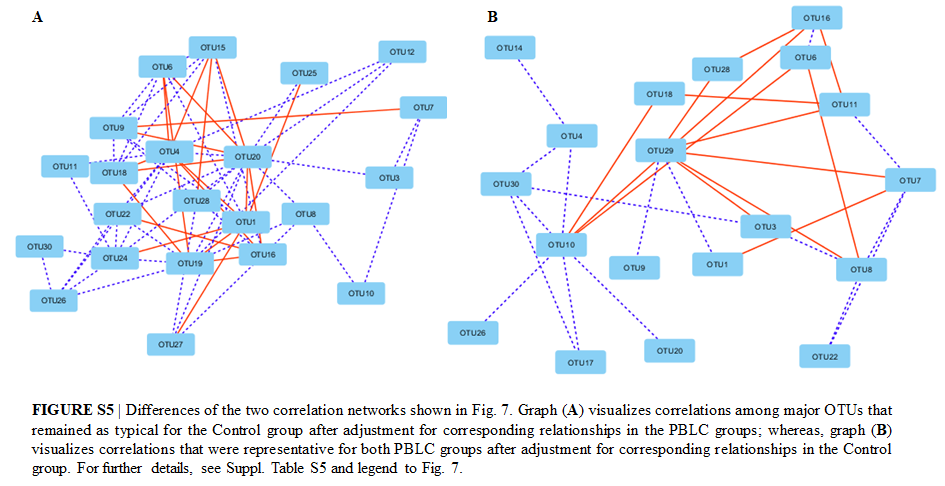

Supplement: Supplementary file 10 [file Image_5.TIF]

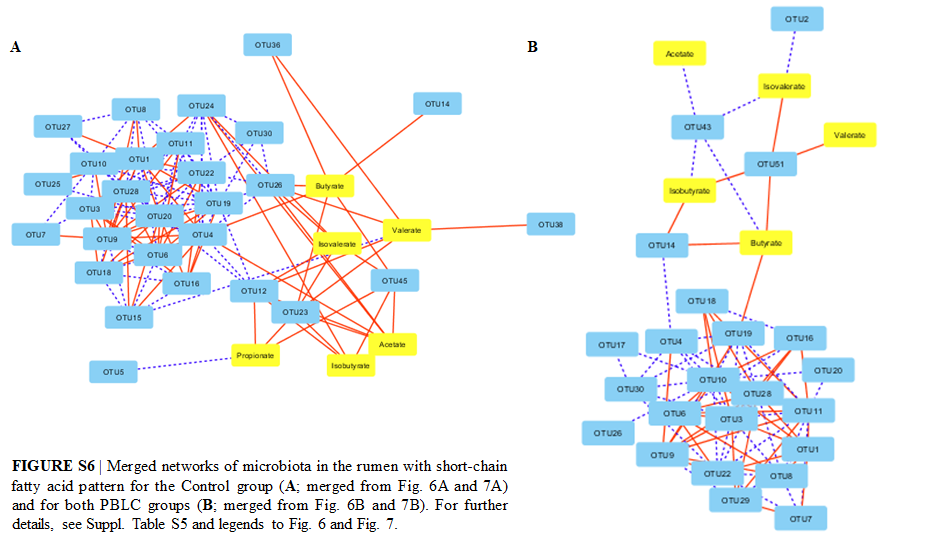

Supplement: Supplementary file 11 [file Image_6.TIF]

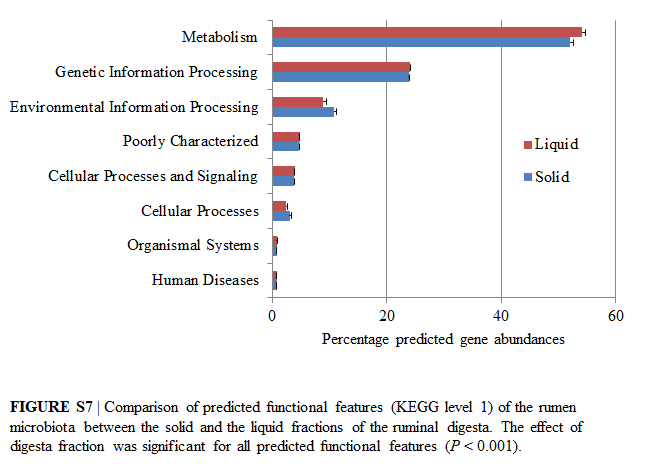

Supplement: Supplementary file 12 [file Image_7.TIF]
